# Novel Roles of the GPI-Anchor Cleaving Enzyme, GDE2, in Hippocampal Synaptic Morphology and Function

**DOI:** 10.1523/ENEURO.0102-25.2025

**Published:** 2025-07-23

**Authors:** Daniel Daudelin, Damani Sama-Borbon, Nan Zhang, Shanthini Sockanathan

**Affiliations:** The Solomon Snyder Department of Neuroscience, The Johns Hopkins School of Medicine, Baltimore, Maryland 21205

**Keywords:** GDE2, hippocampus, morphology, NMDAR-LTD, PI3K signaling

## Abstract

Hippocampal synaptic activity is tightly regulated to ensure appropriate synaptic function and plasticity, which are important for critical cognitive processes such as learning and memory. Altered hippocampal synaptic function can lead to cognitive and behavioral deficits observed in neurodegenerative diseases such as Alzheimer's disease (AD), necessitating a deeper fundamental understanding of hippocampal synaptic control mechanisms. Glycerophosphodiester phosphodiesterase 2 (GDE2 or GDPD5) is a surface transmembrane enzyme that cleaves the glycosylphosphatidylinositol anchor that tethers some proteins to the membrane. Mice lacking GDE2 (*Gde2*KO) display behavioral deficits in learning and memory that are hippocampal-dependent. However, roles of GDE2 in mouse hippocampal function are not known. Here, we show that GDE2 is expressed in pre- and postsynaptic compartments along apical dendrites in hippocampal CA1 cells. *Gde2*KO CA1 cells showed increased dendritic length and complexity and increased numbers of mushroom spines localized to the stratum radiatum. Furthermore, adult *Gde2*KOs displayed an increased frequency of miniature excitatory postsynaptic currents, impaired paired-pulse facilitation, and disrupted *N*-methyl-d-aspartate receptor (NMDAR)-mediated long–term depression (LTD). The phosphatidylinositol 3-kinase-AKT-glycogen synthase kinase 3 (PI3K-AKT-GSK3) signaling pathway, implicated in the inhibition of NMDAR-mediated LTD, was abnormally activated in the *Gde*2KO hippocampus, and inhibition of PI3K restored *Gde2*KO NMDAR-mediated LTD to WT levels. These observations identify GDE2 as an essential physiological regulator of CA1 synaptic morphology and hippocampal pre- and postsynaptic function, including the modulation of NMDAR-mediated LTD via the PI3K-AKT-GSK3 signaling axis.

## Significance Statement

Hippocampal synaptic function is critical for important cognitive functions such as learning and memory. Understanding how hippocampal synaptic activity is regulated could help clarify the basis of diseases such as Alzheimer's disease, where these cognitive functions are impaired. This study identifies a new player that is important for regulating hippocampal synaptic morphology and function. Glycerophosphodiester phosphodiesterase 2 (GDE2) is essential for maintaining normal dendritic structure and synaptic activity in hippocampal CA1 cells. Loss of GDE2 leads to altered synaptic plasticity, including disrupted *N*-methyl-d-aspartate receptor-mediated long–term depression, which is due to aberrant activation of the phosphatidylinositol 3-kinase-AKT-glycogen synthase kinase 3 signaling pathway. These results provide new insight into the molecular mechanisms underlying hippocampal synaptic regulation.

## Introduction

Appropriate regulation of synaptic function is fundamental to communication between neurons, enabling the transmission of signals that underlie cognition and behavior (Citri and Malenka, 2008; Kennedy, 2013). In the hippocampus, a brain region essential for certain forms of learning and memory, synaptic plasticity mechanisms such as paired-pulse facilitation (PPF) and long-term depression (LTD) are crucial for adaptive changes in synaptic signaling (Creager et al., 1980; Dudek and Bear, 1992; Leung and Fu, 1994; Santschi and Stanton, 2003; Citri and Malenka, 2008). PPF is a short-term enhancement of synaptic transmission, while LTD is a prolonged weakening of synaptic responses, both of which are essential for fine-tuning neural circuits (Lynch et al., 1977; Creager et al., 1980; Dudek and Bear, 1992). These mechanisms are integral to memory formation, synaptic efficacy, and homeostatic plasticity, contributing to the encoding, consolidation, and retrieval of information (Hebb, 1949; Nabavi et al., 2014; Jackman and Regehr, 2017; Li et al., 2019).

Synaptic dysfunction is a hallmark feature of several neurodegenerative diseases, contributing to the cognitive and behavioral impairments observed in these conditions (Taylor et al., 2016; Knopman et al., 2021). In Alzheimer's disease (AD), studies in patients and preclinical mouse models reveal synaptic deficits, particularly in the hippocampus, including early-stage neuronal hyperactivity and misregulation of LTD and PPF, ultimately culminating in neurodegeneration and death (Busche et al., 2008; Knopman et al., 2021; Taylor et al., 2021; Meftah and Gan, 2023). Similarly, in animal models and patients with AD and amyotrophic lateral sclerosis–frontotemporal dementia (ALS–FTD), progressive synaptic dysfunction is accompanied by behavioral disruptions that eventually result in fatality, further highlighting the importance of synaptic integrity and regulated activity (Taylor et al., 2016; Knopman et al., 2021; Gelon et al., 2022). One focus of research into potential therapies for these diseases has been on chemical inhibitors targeting molecular pathways that regulate synaptic function with the aim of restoring synaptic health and alleviating symptoms (Brown et al., 2020; Pardo-Moreno et al., 2022). Attaining these goals necessitates a deeper understanding of the fundamental pathways involved in synaptic regulation to identify new targets for therapeutic development. Consequently, understanding the intricate signaling pathways that govern synaptic modulation, particularly in the hippocampus, is critical for unraveling the links between synaptic health and behavioral performance.

Glycerophosphodiester phosphodiesterase 2 (GDE2 or GDPD5) is a member of a small family of three six-transmembrane proteins that act at the cell surface to cleave the glycosylphosphatidylinositol (GPI) anchor that tethers certain proteins to the membrane (Rao and Sockanathan, 2005; Yanaka, 2007; Park et al., 2013). GDE2 is the only family member that is expressed in neurons and is widely expressed in the adult nervous system, including the hippocampus, cortex, thalamus, amygdala, and spinal cord (Cave et al., 2017; Choi et al., 2020, 2021; Daudelin et al., 2024). During development, GDE2 promotes the differentiation of specific spinal and cortical neuronal subtypes and releases neuronal factors to coordinate oligodendrocyte maturation (Rao and Sockanathan, 2005; Sabharwal et al., 2011; Rodriguez et al., 2012). In the adult nervous system, GDE2 is required for neuronal survival via pathways that are distinct from its developmental functions (Cave et al., 2017; Nakamura et al., 2021). Loss of GDE2 recapitulates cellular and behavioral abnormalities observed in AD and ALS–FTD (Nakamura et al., 2021; Westerhaus et al., 2022; Daudelin et al., 2024; Zhang et al., 2024). For example, aged mice lacking GDE2 (*Gde2*KO) exhibit progressive neuronal loss, a reduction in cortical synaptic proteins and synapses, an increase in toxic Aβ42 fragments, and nuclear exclusion and dysfunction of TDP-43 (Nakamura et al., 2021; Zhang et al., 2024). Moreover, *Gde2*KOs display behavioral deficits as early as 7 months of age, including impairments to sociability and short- and long-term memory that are seen in models of AD and ALS/FTD, and motor deficits typical of ALS (Daudelin et al., 2024). Interestingly, GDE2 is found to aberrantly accumulate in neurons of the postmortem brain of patients with AD, ALS–FTD, and ALS, and consistent with disrupted GDE2 function, GPI-anchored protein release is reduced in AD and ALS (Nakamura et al., 2021; Westerhaus et al., 2022). These collective observations suggest that GDE2 loss may contribute to neuropathologies observed in AD, ALS–FTD, and ALS. While GDE2 activity is disrupted in neurodegenerative diseases affecting cognition, and *Gde2*KO mice display behavioral deficits associated with hippocampal dysfunction (Nakamura et al., 2021; Westerhaus et al., 2022; Daudelin et al., 2024), the role of GDE2 in the hippocampus has not been addressed. Here we sought to test the hypothesis that GDE2 affects hippocampal synaptic function and morphology.

## Materials and Methods

### Animals

All animal procedures were performed in accordance with the Johns Hopkins University animal care committee's regulations. *Gde2*KO and WT mice were bred, maintained, and genotyped as described previously (Sabharwal et al., 2011). Both male and female mice were used for analysis. Mice were group-housed using a standard 12 h light/dark cycle.

### Synaptosome preparation

Synaptosomes were prepared according to established protocols (Dunkley et al., 2008). Briefly, homogenizing/dissecting buffer and Percoll gradient solutions were prepared the day of or night before dissection. For one dissection, 50 ml of homogenizing/dissecting buffer was made [12.5 ml of 4X SET buffer (1.28 M sucrose, 4 mM EDTA, 20 mM Tris), 0.25 ml of 50 mM dithiothreitol (DTT), and 37.25 ml of nuclease free H_2_O], pH 7.4. Ten milliliters each of 23, 15, 10, and 3% Percoll solutions (Sigma-Aldrich P1644; 2.3, 1.5, 1, and 0.3 ml Percoll, respectively) were made with 2.5 ml of 4× SET buffer, 0.05 ml of 50 mM DTT, and the appropriate amount of nuclease-free H_2_O (5.15, 5.95, 6.45, and 7.15 ml, respectively). All solutions were filtered through a 0.2 μm syringe filter and vortexed. Before dissection, protease (Sigma-Aldrich, 11873580001) and phosphatase (Sigma-Aldrich, 4906845001) inhibitors were added to a homogenizing buffer (2.25 ml), which was chilled on ice and used for both hippocampi from one brain. The dissection buffer was frozen to slush before being used for dissection.

Hippocampi were slowly homogenized in the homogenizing buffer on ice using 30 strokes of a Dounce homogenizer, and the homogenate was centrifuged at 1,000 × *g* for 10 min at 4°C. The supernatant (S1) was transferred to a 13 ml ultracentrifuge tube preloaded with a gradient of 2.5 ml of 23, 15, 10, and 3% Percoll solutions and centrifuged at 32,800 × *g* for 7 min. The synaptosome fraction between 15 and 23% was collected, a 4× gel Laemmli buffer was added, and Western blots were performed.

### AAV vector and immunohistochemistry (IHC)

The adeno-associated retrovirus (AAV) plasmid backbone was based on AAV-GFP/Cre (RRID:Addgene_49056). The coding sequence for mGDE2-cFLAG was synthesized and subcloned into the AAV backbone by replacing GFP/Cre. The AAV vectors were packaged by Janelia Viral Tools using the PHP.eB capsid (Deverman et al., 2016; Chan et al., 2017). AAV vectors were delivered to mice retro-orbitally at p28 at 10^11^ viral genome per animal as previously described (Chan et al., 2017). Animals were anesthetized using 0.01 ml/g Avertin [1.3% 2,2,2-tribromoethanol (T48402-25G) and 0.7% 2-methyl-2-butanol (Sigma-Aldrich 240486) in phosphate-buffered saline (PBS)] prior to injection. Animals were monitored postinjection, and tissues were harvested at time points indicated.

The tissue was prepared for IHC as previously described (Zhang et al., 2024). Briefly, mice were anesthetized with 0.02 ml/g Avertin solution and 0.7% 2-methyl-2-butanol (Sigma-Aldrich 240486) in PBS before transcardial perfusion with a 0.1 M phosphate buffer (PB) and 4% paraformaldehyde (PFA) in 0.1 M PB. Brains were dissected, postfixed in 4% PFA for 18–20 h, washed with PBS, and prepared for embedding in cryomolds. Slices were sectioned at 40 µm on a Leica CM3050 S cryostat and IHC-immunofluorescence was performed using free-floating sections. Sections were washed in PBS before permeabilization in 0.3% Triton X-100 in PBS (PBST) followed by a 2 h block in 5% bovine serum albumin (BSA; Sigma-Aldrich A9647) in PBST at room temperature (RT). Following blocking, tissue sections were incubated with primary antibodies against DYKDDDDK Tag (Flag, Cell Signaling Technology, #14793S, 1:1,000; RRID:AB_2572291), postsynaptic density-95 (PSD-95; Invitrogen, #MA1-046, 1:500; RRID:AB_2092361), and Bassoon (Abcam, #ab82958, 1:500; RRID:AB_1860018) at 4°C overnight. After PBS washes, sections were incubated with fluorescent-conjugated secondary antibodies (AF488; RRID:AB_2943314, AF568; RRID:AB_2943328) as well as Hoechst 33342 (Thermo Fisher Scientific, 62249, 1:1,000) for at least 2 h at RT. After PBS washes, coverslips were mounted on Superfrost Plus microscope slides in Prolong Diamond Antifade Mountant (Thermo Fisher Scientific, P36970). All images were then acquired using a Zeiss LSM700 microscope or Zeiss LSM880 microscope with Airyscan super-resolution technology for increased resolution in all three axes (*x*, *y*, *z*). All images acquired using the Airyscan had a detector at 63× with 1.8 zoom with a *z*-step of 0.3 μm.

### Immunocytochemistry (ICC)

Mouse primary hippocampal cultures were prepared from postnatal day (P)0 or P1 mouse hippocampi and plated on poly-l-lysine–coated and acid-washed coverslips as previously described (Nakamura et al., 2021) with minor modifications. Cells were maintained in Neurobasal medium supplemented with 2% B27, 1% l-glutamine, and 1% pen/strep. In total, 5 µM cytosine arabinoside was added on day in vitro (DIV)2 to inhibit glial growth and removed on DIV3, followed by half media changes every 3 d.

Cultures were transduced with AAV mGDE2-cFlag at an MOI of 10,000 at DIV11. Cultures were maintained at 37°C until harvest at DIV14. At DIV14, cells were fixed with 4% PFA at 4°C for 12 min. Fixed cells were then permeabilized with 0.3% PBST and blocked with 5% BSA in PBST for at least 1 h. Cells were then incubated with primary antibodies against DYKDDDDK Tag (Flag, Cell Signaling Technology, #14793S, 1:1,000; RRID:AB_2572291), PSD-95 (Invitrogen, #MA1-046, 1:1,000; RRID:AB_2092361), Bassoon (Abcam, #ab82958, 1:1,000; RRID:AB_1860018), and MAP2 (Synaptic Systems, #188004, 1:5,000; RRID:AB_2138181), at 4°C overnight. After PBST washes, cells were incubated with fluorescent-conjugated secondary antibodies (AF488; RRID:AB_2943314, AF568; RRID:AB, AF647; RRID:AB_2943328) as well as Hoechst 33342 (Thermo Fisher Scientific, 62249, 1:1,000) for at least 1 h at RT. After PBST and PBS washes, coverslips were mounted on Superfrost Plus microscope slides in Prolong Diamond Antifade Mountant (Thermo Fisher Scientific, P36970). All images were then acquired using a Zeiss LSM880 microscope with Airyscan super-resolution technology, for increased resolution in all three axes (*x*, *y*, *z*). All images were acquired using the Airyscan detector at 63× with 1.8 zoom with a *z*-step of 0.3 μm.

### Quantitative image analysis (IHC/ICC)

After image acquisition, raw Airyscan images from the LSM880 were processed into a useable form in the Zeiss Zen Blue 3.1 analysis suite. Colocalization analysis was done in the ImageJ suite (NIH) using the JaCoP plugin (Bolte and Cordelieres, 2006). For the mouse tissue, at least three sections were analyzed per mouse, with three regions of interest (ROIs) chosen at random per section. For in vitro experiments, the neurites of at least 18 MAP2+ cells per biological sample were analyzed. Neurites were defined as the region within 0.5 μm of the MAP2 stain.

### Golgi–Cox staining and analysis of hippocampal CA1 apical dendrites and spines

Golgi–Cox staining was conducted using the FD Rapid GolgiStain Kit (FD NeuroTechnologies), following the manufacturer's protocol with minor modifications. Briefly, mice were anesthetized with CO_2_ and decapitated. The brains were quickly removed and rinsed in double-distilled water. Forebrains and cerebellum were removed, halved and submerged in 3.5 ml of an impregnation solution (1:1 mixture of Solutions A and B, prepared 24 h prior) at RT for 10 d, with the solution replaced after the first day. Subsequently, the tissue was transferred to 4 ml of solution C at RT for 1 week. Finally, the tissue was frozen in isopentane cooled with dry ice using a plastic spoon.

The frozen tissue was sectioned into 100 μm slices using a cryostat and immediately mounted on gelatin-coated microscope slides using solution C. After air-drying overnight at RT and two 4 min washes with double-distilled water, the sections were incubated in a working solution (1:1:6 ratio of Solutions D, E, and double-distilled water) with gentle shaking for 8.5 min. Following two 4 min washes in Milli-Q water, the sections were dehydrated through a graded series of ethanol (50, 75, 95, and 100% I, II, III for 5 min each), cleared in xylene (three 10 min washes), and coverslipped with Eukitt Quick-hardening mounting medium. The sections were shielded from light as much as possible throughout the processing. Neurolucida (MBF Bioscience) was used to create 3D tracings of the morphologies of CA1 dendrites and spines using a 100× lens on a Zeiss light microscope. Neuroexplorer (MBF Bioscience) was used for quantification and Sholl analysis.

### Hippocampal slice preparation for electrophysiology and PI3K inhibition

Three hundred micrometer (for whole-cell patch–clamp recordings) and 400 μm (for extracellular field recordings) thick coronal slices containing hippocampal sections were prepared according to previous protocols (Ting et al., 2018; Levy-Myers et al., 2023). Briefly, male and female mice aged 1.5- and 7 months were used for acute-slice electrophysiology recording experiments. For dissecting the brain and for perfusion, a chilled, oxygenated (95% O_2_ and 5% CO_2_) dissection solution was used (in mM: 92 *N*-methyl-d-glucamine, 2.5 KCl, 1.25 NaH_2_PO_4_, 30 NaHCO_3_, 20 HEPES, 25 glucose, 2 thiourea, 5 sodium ascorbate, 3 sodium pyruvate, 0.5 CaCl_2_·2H_2_O, and 10 MgSO_4_·7H_2_O), pH 7.3 to pH 7.4. After dissection and slicing by vibratome (Leica VT1200 S), slices were transferred to 40 ml of the same solution for 25 min at 32°C. During this interval, a 2 M NaCl spike-in solution was added to the solution in 5 min intervals as follows: 66 μl as the slices were added and at 5 min, 133 μl at 10 min, 266 μl at 15 min, and 533 μl at 20 min, giving a final concentration of 92 mM NaCl. Afterward, slices were transferred to 100 ml of RT holding artificial cerebrospinal fluid (hACSF; in mM: 92 NaCl, 2.5 KCl, 1.25 NaH_2_PO_4_, 30 NaHCO_3_, 20 HEPES, 25 glucose, 2 thiourea, 5 sodium ascorbate, 3 sodium pyruvate, 2 CaCl_2_·2H_2_O, and 2 MgSO_4_·7H_2_O), pH 7.3–7.4. Slices were incubated for at least 1 h in the hACSF before patch-clamp recordings and at least 2 h in hACSF before field recording experiments. For PI3K rescue experiments, 10 μM LY294002 was added to the hACSF.

### Whole-cell patch–clamp and field recording electrophysiology

Recordings and subsequent analysis were carried out blind to genotype. Hippocampal CA1 pyramidal cells just below the slice surface were visualized using infrared differential interference contrast optics. When a healthy cell was located, a borosilicate pipette (2–4 MΩ) was used for recording. Voltage-clamp recordings were conducted using a MultiClamp 700B amplifier (Molecular Devices), and slices were immersed in recirculated, aerated (95% O_2_ and 5% CO_2_) extracellular recording ACSF [124 mM NaCl, 2.5 mM KCl, 1.25 mM NaH_2_PO_4_, 24 mM NaHCO_3_, 5 mM HEPES, 12.5 mM glucose, 2 mM CaCl_2_·2H_2_O, 2 mM MgSO_4_·7H_2_O, 1 μM tetrodotoxin (TTX; Abcam ab120054), and 40 μM bicuculline (Sigma-Aldrich, 14343)], pH 7.3–7.4, held at 31°C.

Recordings were conducted using an internal solution (in mM: 135 CsMeSO_3_, 10 CsCl, 10 HEPES, 0.2 EGTA, 3 Na_2_ATP, 0.3 Na_3_GTP, and 5 QX 314), pH 7.3–7.4, while the voltage of the cells was clamped at −60 mV. Data were recorded and analyzed using pCLAMP 10 (Molecular Devices), Wdetecta, and Wscnslct (Huguenard Laboratory). After successfully patching a cell, a 5 min equilibrium period was allowed to elapse, followed by 10 min of data collection. We measured access and membrane resistances throughout, and cells where access resistances were over 25 MΩ or membrane resistances were under 150 MΩ at any time were excluded from the analysis. A 5 pA amplitude cutoff was used as an event threshold. The automated software detection system initially isolated events, and subsequently, nonevents caused by noise were excluded upon visual inspection of the traces. Cells with <50 events were not included in the final analysis. Over 200 events for both genotypes were averaged together to construct representative traces. Mean amplitude and frequency bar graphs were created by averaging all events for each cell. Fifty random events per cell were used to construct cumulative probability plots to avoid bias from more active cells.

Slices for field recordings were prepared as described above and as previously described (Delgado et al., 2007). Briefly, slices were allowed to recover in RT ACSF for 2 h before recording. For recording, slices were maintained in a recording chamber with temperature held constant at 31°C, and recording ACSF (without TTX or bicuculline) was recirculated while being aerated (95% O_2_ and 5% CO_2_). Schaffer collaterals were activated using a bipolar, nichrome wire electrode placed in the stratum radiatum, and synaptic potentials were recorded using glass microelectrodes filled with ACSF also in the stratum radiatum (Fig. 4*A*). During LTD experiments, test pulses (0.2 ms in duration) were delivered every 30 s during baseline acquisition (30 min of stable responses) and 1 h post induction. Two different protocols were used for LTD induction: for *N*-methyl-d-aspartate receptor (NMDAR)-induced LTD 1 Hz low-frequency stimulation (LFS; 900 pulses) was used, while for mGluR-LTD, 100 μM *S*-DHPG (Tocris Bioscience, 8050) was applied for 15 min. For PPR experiments, 15 sets of paired pulses (30 s apart) were delivered separated by 25, 50, 100, 200, 400, 1,000, and 2,000 ms sequentially. Data were collected and analyzed with pCLAMP 10 (Molecular Devices).

### Immunoblotting

Western blots were conducted according to published protocols (Zhang et al., 2024). Briefly, the hippocampal tissue was sonicated in a RIPA buffer (10 mM Tris–HCl, 1 mM EDTA, 0.5 mM EGTA, 1% Triton X-100 • 0.1% sodium deoxycholate, 0.1% SDS, 140 mM NaCl, diluted with d H_2_O), pH 8.0, containing 1× protease and phosphatase inhibitor cocktail (Sigma-Aldrich, PPC1010) and centrifuged at 21,000 × *g* for 20 min at 4°C. Protein concentrations for all samples were standardized using a BCA Protein Assay kit (Thermo Fisher Scientific, 23,225), and a 4× Laemmli buffer was added to yield a final concentration of 1×.

Samples in a Laemmli buffer were boiled and run on 7.5 or 10% polyacrylamide gels in a Tris/glycine running buffer before transferring to a polyvinylidene difluoride membrane using chilled transfer buffer (25 mM Tris, 192 mM glycine, 10% methanol). After transfer, membranes were blocked with 5% milk in Tris-buffered saline containing 0.3% Tween-20 (TBST) or EveryBlot blocking buffer (Bio-Rad Laboratories, 12010020) for 1 h at RT. Following blocking, primary antibodies were applied overnight at 4°C. After TBST washes, membranes were incubated for 1 h at RT with horseradish peroxidase or fluorescent protein-conjugated secondary. Following a final set of TBST washes, membranes were developed using enhanced chemiluminescence substrate when needed (Kindle Biosciences, R1004), imaged using a ChemiDoc Imager (Bio-Rad Laboratories) or KwikQuant Imager (Kindle Biosciences), and analyzed using the ImageJ software (NIH). The following antibodies were used at the following concentrations: GDE2 (Covance, 1:1,000; Nakamura et al., 2021), neuronal pentraxin receptor (NPTXR; R&D Systems, #AF4414; RRID: AB_2153869, 1:1,000), glutamate ionotropic receptor NMDA type subunit 1 (GluN1; a gift from R. Huganir, #JH4440, 1:1,000), glutamate ionotropic receptor alpha-amino-3-hydroxy-5-methyl-4-isoxazolepropionic acid Type 1 subunit (GluA1; a gift from R. Huganir, #4.9D, 1:1,000), PSD-95 (Invitrogen, #MA1-046, 1:1,000; RRID:AB_2092361), synaptophysin (Sigma-Aldrich, #S5768, 1:1,000; RRID:AB_477523), Homer (Santa Cruz Biotechnology, #SC-17842, RRID:AB_627742), AKT (Cell Signaling Technology, #9272, 1:10,000; RRID:AB_329827), pAKT(S473) (Cell Signaling Technology, #4060, 1:1,000; RRID:AB_2315049), pAKT(T308) (Cell Signaling Technology, #9275, 1:500; RRID:AB_329828), GSK3α/β (Cell Signaling Technology, #5676, 1:10,000; RRID:AB_10547140), pGSK3α/β (Cell Signaling Technology, #9331, 1:1,000; RRID:AB_329830), S6 (Santa Cruz Biotechnology, #sc-74459,1:1,000, RRID:AB_1129205), pS6(S240/244) (Cell Signaling Technology, #5364, 1:1,000, RRID:AB_10694233), Actin (Millipore, #MAB1501R, 1:10,000, RRID:AB_2223041), and Actin(Rhodamine) (Bio-Rad Laboratories, #12004163, 1:2,000; RRID:AB_2861334).

### Statistics

Data were analyzed and plotted with GraphPad Prism (version 10) with a minimum significance level of *p* < 0.05. Two-tailed Student's *t* test with appropriate corrections was used to determine the significance of pairwise comparisons. For comparisons between more than two groups, ANOVA with corrections for multiple comparisons was used. In all cases, data were tested for normal distribution. For colocalization analysis, Mander's coefficients were used, calculated as the proportion of intensities of Channel 1 that coincide with Channel 1. The coefficient ranges from 0 to 1, with 0 indicating no colocalization and 1 indicating 100% colocalization. For example, a Mander’s coefficient for Protein A: Protein B of 0.2 would mean that 20% of Protein A in an ROI is colocalized with Protein B. Two tests were used to ensure proper colocalization: (1) Van Steensel's x-shift (van Steensel et al., 1996), which shifts one image relative to the other pixel by pixel (δx) to test if maximal colocalization occurs at δx = 0, and (2) Costes randomization (Costes et al., 2004), which randomizes the pixels in each image to obtain a normal distribution to compare the experimental colocalization coefficient. Data are reported as mean ± SEM. In figures, asterisks denote the following statistical significance: *p < 0.05, **p < 0.01, ***p < 0.001, and ****p < 0.0001. Statistical information for experiments is included in figure legends and Table 1.

**Table 1.** Statistics table

	Data structure	Type of test	Power
Figure 1*G*	Normal distribution	Welch's *t* test	*p* < 0.0001
Figure 1*H*	Normal distribution	Welch's *t* test	*p* = 0.0373
Figure 1*J*	Normal distribution	Welch's *t* test	*p* < 0.0001
Figure 1*K*	Normal distribution	Welch's *t* test	*p* = 0.0097
Figure 2*D*	Normal distribution	Unpaired *t* test	*p* = 0.0124
Figure 2*E*	Normal distribution	Unpaired *t* test	*p* = 0.0165
Figure 2*F*	Normal distribution	Unpaired *t* test	*p* = 0.0003
Figure 2*G*	Normal distribution	Unpaired *t* test	*p* = 0.0878
Figure 2*H*	Normal distribution	Unpaired *t* test	*p* = 0.8528
Figure 2*I*	Normal distribution	Unpaired *t* test	*p* < 0.0001
Figure 2*J*	Normal distribution	Unpaired *t* test	*p* = 0.1009
Figure 2*K*	Normal distribution	Unpaired *t* test	*p* = 0.3619
Figure 2*L*	Normal distribution	Unpaired *t* test	*p* = 0.1643
Figure 2*M*	Normal distribution	Unpaired *t* test	*p* = 0.0330
Figure 2*N*	Normal distribution	Unpaired *t* test	*p* < 0.0001
Figure 2*O*	Normal distribution	Two-way repeated–measure ANOVA	Radius, 100 µm, *p* = 0.0003; radius, 120 µm, *p* = 0.0066
Figure 2*P*	Normal distribution	Two-way repeated–measure ANOVA	Radius, 80 µm, *p* = 0.0026; radius, 100 µm, *p* = 0.0173, radius, 120 µm, *p* = 0.0026
Figure 2*R*	Normal distribution	Two-way repeated–measure ANOVA	Radius, 100 µm, *p* = 0.0007; radius, 120 µm, *p* = 0.0220
Extended Data Figure 2-1*B*	Normal distribution	Unpaired *t* test	*p* = 0.2126
Extended Data Figure 2-1*D*	Normal distribution	Unpaired *t* test	*p* = 0.6036
Extended Data Figure 2-1*F*	Normal distribution	Unpaired *t* test	*p* = 0.1765
Figure 3*B*	Normal distribution	Unpaired *t* test	*p* = 0.0976
Figure 3*C*	Normal distribution	Kolmogorov–Smirnov test	*p* = 0.0009
Figure 3*E*	Normal distribution	Unpaired *t* test	*p* = 0.0223
Figure 3*F*	Normal distribution	Kolmogorov–Smirnov test	*p* < 0.0001
Figure 3*G*	Normal distribution	Two-way repeated–measure ANOVA	ISI, 25 ms, *p* < 0.0001; ISI, 50 ms, *p* = 0.0034
Figure 3*I*	Normal distribution	Unpaired *t* test	*p* = 0.0085
Extended Data Figure 3-1*A*	Normal distribution	Unpaired *t* test	*p* = 0.5239
Extended Data Figure 3-1*B*	Normal distribution	Unpaired *t* test	*p* = 0.9496
Extended Data Figure 3-1*C*	Normal distribution	Unpaired *t* test	*p* = 0.6225
Extended Data Figure 3-1*D*	Normal distribution	Unpaired *t* test	*p* = 0.7059
Extended Data Figure 3-1*F*	Normal distribution	Unpaired *t* test	*p* = 0.0790
Extended Data Figure 3-1*G*	Normal distribution	Kolmogorov–Smirnov test	*p* < 0.0001
Extended Data Figure 3-1*I*	Normal distribution	Unpaired *t* test	*p* = 0.0949
Extended Data Figure 3-1*J*	Normal distribution	Kolmogorov–Smirnov test	*p* < 0.0001
Extended Data Figure 3-1*K*	Normal distribution	Unpaired *t* test	*p* = 0.2225
Extended Data Figure 3-1*L*	Normal distribution	Unpaired *t* test	*p* = 0.9490
Extended Data Figure 3-1*M*	Normal distribution	Unpaired *t* test	*p* = 0.4825
Extended Data Figure 3-1*N*	Normal distribution	Unpaired *t* test	*p* = 0.4013
Extended Data Figure 3-2*B*	Normal distribution	Unpaired *t* test	*p* < 0.0001
Extended Data Figure 3-3*D*	Normal distribution	Unpaired *t* test	*p* = 0.4783
Extended Data Figure 3-3*F*	Normal distribution	Unpaired *t* test	*p* = 0.8145
Figure 4*E*	Normal distribution	Unpaired *t* test	*p* = 0.0016
Figure 4*F*	Normal distribution	Unpaired *t* test	*p* = 0.0386
Figure 4*G*	Normal distribution	Unpaired *t* test	*p* = 0.0119
Figure 4*H*	Normal distribution	Unpaired *t* test	*p* = 0.0120
Extended Data Figure 4-1*A*	Normal distribution	Unpaired *t* test	*p* = 0.5782
Extended Data Figure 4-1*B*	Normal distribution	Unpaired *t* test	*p* = 0.6481
Extended Data Figure 4-1*C*	Normal distribution	Unpaired *t* test	*p* = 0.5931
Extended Data Figure 4-1*D*	Normal distribution	Unpaired *t* test	*p* = 0.2083
Extended Data Figure 4-1*F*	Normal distribution	Unpaired *t* test	*p* = 0.3130
Extended Data Figure 4-1*G*	Normal distribution	Unpaired *t* test	*p* = 0.3850
Extended Data Figure 4-2*B*	Normal distribution	Unpaired *t* test	*p* = 0.0033
Extended Data Figure 4-2*C*	Normal distribution	Unpaired *t* test	*p* = 0.2173
Extended Data Figure 4-2*E*	Normal distribution	Unpaired *t* test	*p* = 0.1375
Extended Data Figure 4-2*F*	Normal distribution	Unpaired *t* test	*p* = 0.3509
Extended Data Figure 4-2*G*	Normal distribution	Unpaired *t* test	*p* = 0.1155
Extended Data Figure 4-2*H*	Normal distribution	Unpaired *t* test	*p* = 0.3224
Figure 5*B*	Normal distribution	One-way ANOVA	WT vs *Gde2*KO, *p* = 0.0118; *Gde2*KO vs *Gde2*KO + LY294002, *p* = 0.0496; WT vs *Gde2*KO + LY294002, *p* = 0.8445
Figure 5*C*	Normal distribution	Two-way repeated–measure ANOVA	ISI, 25 ms, WT vs *Gde2*KO, *p* = 0.0010; WT vs *Gde2*KO + LY294002, *p* = 0.0005; ISI, 50 ms, WT vs *Gde2*KO, *p* = 0.0223; WT vs *Gde2*KO + LY294002, *p* = 0.0229
Figure 5*E*	Normal distribution	One-way ANOVA	WT vs *Gde2*KO, *p* = 0.0011; *Gde2*KO vs *Gde2*KO + LY294002, *p* < 0.0001; WT vs *Gde2*KO + LY294002, *p* = 0.0156
Figure 5*F*	Normal distribution	One-way ANOVA	WT vs *Gde2*KO, *p* = 0.0140; *Gde2*KO vs *Gde2*KO + LY294002, *p* < 0.0013; WT vs *Gde2*KO + LY294002, *p* = 0.2668
Figure 5*H*	Normal distribution	One-way ANOVA	WT vs *Gde2*KO, *p* = 0.0059; *Gde2*KO vs *Gde2*KO + LY294002, *p* = 0.0068; WT vs *Gde2*KO + LY294002, *p* = 0.9942
Figure 5*I*	Normal distribution	One-way ANOVA	WT vs *Gde2*KO, *p* = 0.0051; *Gde2*KO vs *Gde2*KO + LY294002, *p* = 0.0077; WT vs *Gde2*KO + LY294002, *p* = 0.9558
Extended Data Figure 5-1*B*	Normal distribution	Unpaired *t* test	*p* = 0.7193
Extended Data Figure 5-1*C*	Normal distribution	One-way ANOVA	*p* > 0.05
Extended Data Figure 5-1*D*	Normal distribution	One-way ANOVA	*p* > 0.05
Extended Data Figure 5-1*E*	Normal distribution	One-way ANOVA	*p* > 0.05

Table summarizing statistical tests used and *p* values for all experiments. Values are also listed in figure legends.

## Results

### GDE2 is localized pre- and postsynaptically in the hippocampus

**Figure 1.**
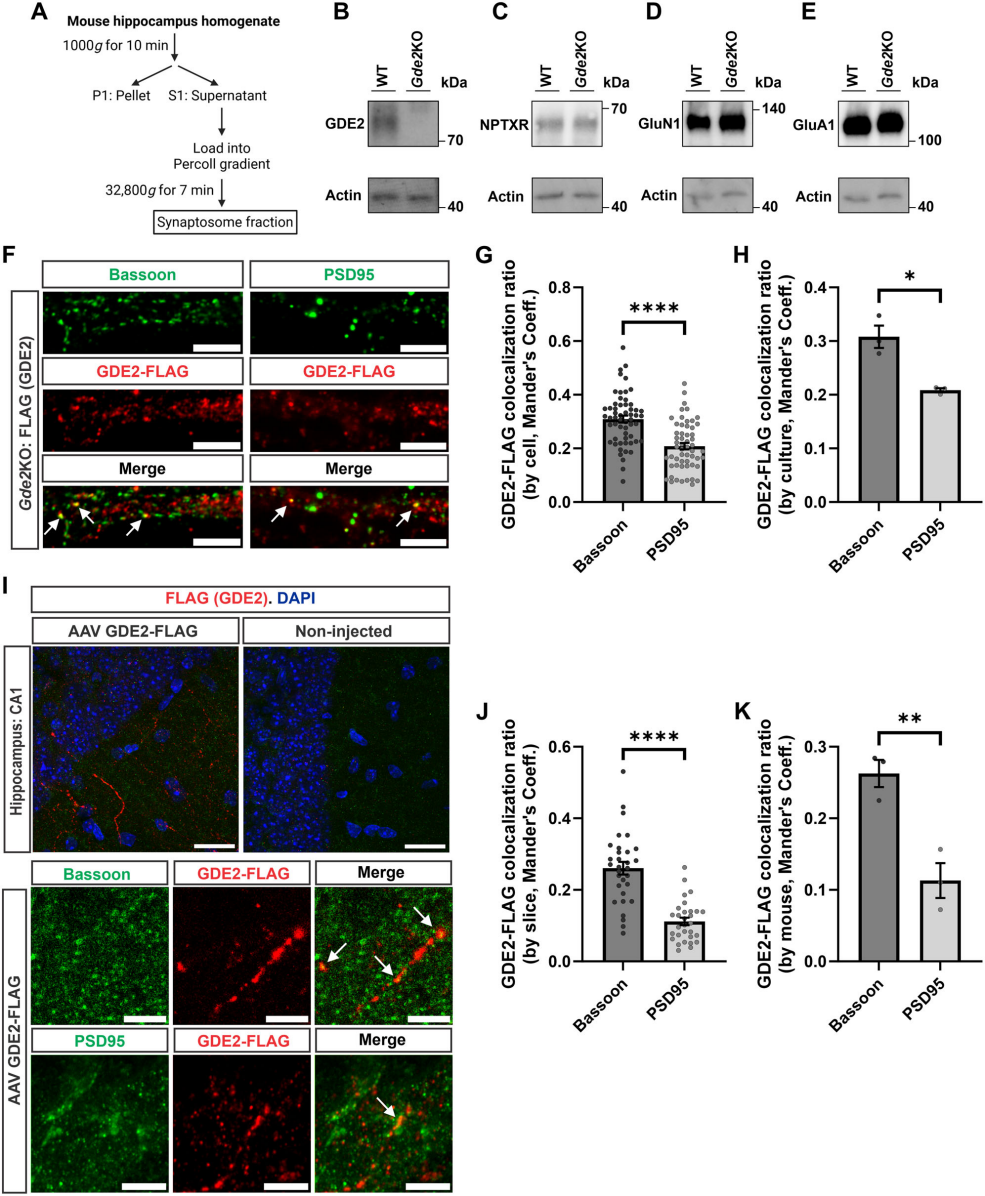
GDE2 is localized pre- and postsynaptically. ***A***, Schematic of synaptosome isolation procedure. ***B*–*E***, Western blots of hippocampal synaptosome lysate from 2-month-old WT and *Gde2*KO mice probing for GDE2 (***B***), NPTXR (***C***), GluN1 (***D***), and GluA1 (***E***). ***F***, Representative images of immunostained cultured primary hippocampal *Gde2*KO neurons transfected with GDE2-FLAG plasmids (Scale bar, 5 μm). Arrows highlight GDE2 colocalization with Bassoon and PSD-95. ***G***, ***H***, Graphs quantifying the ratio of GDE2-FLAG colocalized with Bassoon and PSD-95 (*n* = 57 neurons (Bassoon), 56 neurons (PSD-95), from 3 cultures, mean ± SEM, Welch's *t* test; ***G*** (analyzed by cell), ****p < 0.0001; ***H*** (analyzed by culture), *p = 0.0373). GDE2-FLAG, Bassoon = 0.31 (31% of GDE2-FLAG expression colocalizes with Bassoon); GDE2-FLAG, PSD-95 = 0.21 (21% of GDE2-FLAG expression colocalizes with PSD-95). ***I***, Representative images of immunostained hippocampal sections in the CA1 region of noninjected and AAV GDE2-FLAG injected 1.5-month-old WT mice [Scale bar, 20 μm (top 2 images), 5 µm (bottom 6 images)]; arrows highlight apical dendrites showing GDE2 colocalization with Bassoon and PSD-95. ***J***, ***K***, Graphs quantifying the ratio of GDE2-FLAG colocalized with Bassoon and PSD-95 (*n* = 31 neurons (Bassoon), 30 neurons (PSD-95) across 3 animals per condition, mean ± SEM, Welch's *t* test; ***J*** (analyzed by slice), ****p < 0.0001; ***K*** (analyzed by mouse), *p = 0.0097). GDE2-FLAG, Bassoon = 0.27 (27% of GDE2-FLAG expression colocalizes with Bassoon); GDE2-FLAG, PSD-95 = 0.098 (9.8% of GDE2-FLAG expression colocalizes with PSD-95). Schematic in ***A*** is created in BioRender.com. See Table 1 for statistical summaries. See Extended Data Figure 1-1 for more details.

Synaptosome isolation is an established method that enriches pre- and postsynaptic components from prepared homogenate (Dunkley et al., 2008). To determine if GDE2 is located in synapses, we isolated hippocampal synaptosomes from 2-month-old WT and *Gde2*KO mice (Fig. 1*A*) and analyzed them by Western blot. WT and *Gde2*KO synaptosomes contained the synaptic protein NPTXR and postsynaptic proteins GluN1 and GluA1 confirming synapse isolation by this method (Fig. 1*C*–*E*). Probing with antibodies against GDE2 detected GDE2 expression in WT synaptosomes but not in *Gde2*KO synaptosomes (Fig. 1*B*). These data suggest that GDE2 is present at hippocampal synapses.

10.1523/ENEURO.0102-25.2025.f1-1 Figure 1-1 **Colocalization randomization and x-shift test representative data** Representative graphs generated from Bassoon/GDE2-Flag images from Figure 1F. For both A and B, a gaussian curve is fitted to the data. (A) Coste’s Method: Plotted data (Black dots) and fitted model (Blue curve) of the distribution of Pearson’s Coefficients (PC, denoted as r) between Bassoon and GDE2-Flag for a single representative z-stack of a neurite, obtained by scrambling the particles in the image 10,000 times, along with the measured Pearson’s Coefficient from the original image (Red line). In this case, the distribution shows that the probability of obtaining the measured PC (Red line) randomly is extremely small, meaning this colocalization is significantly above chance, and therefore can be used in analysis. (B) Van Steensel’s x-shift: Plotted data (Black) and fitted model (Blue curve) of the cross-correlation function (CCF, as measured by PC) between Bassoon and GDE2-Flag for a single representative z-stack of a neurite as a function of the pixel shift (dx) between the stains in the positive and negative direction. The red line delineates the PC in the original image. As expected, a larger pixel shift between the stains leads to a smaller correlation, indicating that our measured PC is unique. Download Figure 1-1, TIF file.

We next determined whether GDE2 is localized to specific synaptic compartments in hippocampal neurons. We generated AAV expressing a form of GDE2 that is tagged at the C-terminal intracellular domain with a FLAG epitope (GDE2-FLAG). Previous studies confirm that tagging GDE2 at the C-terminal domain does not interfere with its expression level, its targeting to the cell surface, and its GPI-anchor cleavage activity (Park et al., 2013). We transfected *Gde2*KO primary cultured neurons at DIV6 with AAV GDE2-FLAG and harvested neurons for analysis at DIV14. Confocal analysis using Airyscan super-resolution imaging revealed that GDE2-FLAG colocalized with the presynaptic protein Bassoon and less so with the postsynaptic protein PSD-95 (Fig. 1*F*–*H*). Analysis of publicly available RNAseq datasets combined with RNAscope studies shows that GDE2 is expressed in CA1 pyramidal cells of the hippocampus (Yao et al., 2021; Daudelin et al., 2024). Accordingly, we retro-orbitally injected 1-month-old WT mice with AAV GDE2-FLAG, harvested the hippocampal tissue 2 weeks postinjection, and analyzed GDE2-FLAG expression in CA1 cells in hippocampal sections by confocal imaging with Airyscan super-resolution technology. GDE2-FLAG was expressed throughout hippocampal CA1 apical dendritic branches in injected mice, with no FLAG expression detected in noninjected animals (Fig. 1*I*). In line with our in vitro experiments, GDE2-FLAG colocalized with Bassoon and, to a lesser extent, with PSD-95 (Fig. 1*I–K*). Costes randomization and Van Steensel's x-shift were used to test for proper colocalization (Extended Data Fig. 1-1; see Materials and Methods for details). Altogether, these experiments suggest that GDE2 is localized pre- and postsynaptically, with potential enrichment in presynaptic compartments in hippocampal neurons.

### GDE2 regulates hippocampal CA1 morphology

**Figure 2.**
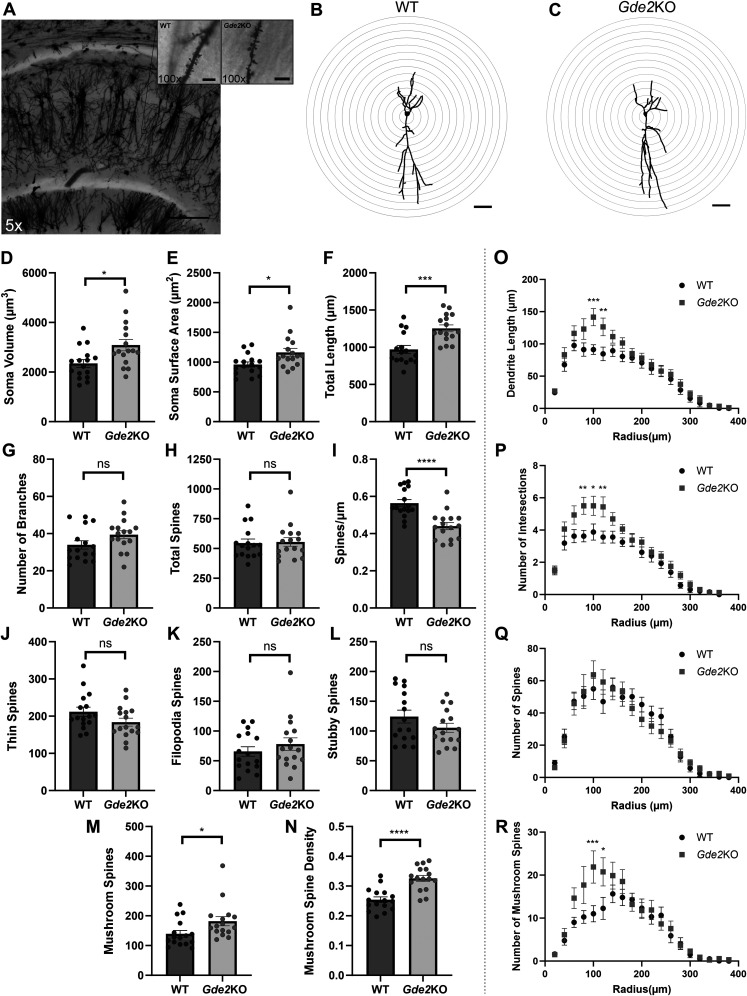
GDE2 regulates the morphology of hippocampal CA1 pyramidal cells. ***A***, Representative image of the Golgi–Cox-stained CA1 region, with insets highlighting apical dendrites with spines (scale bar, 200 µm; inset, 5 µm). ***B***, ***C***, Representative 3D tracing of WT (***B***) and *Gde2*KO (***C***) neurons with concentric circles increasing in radius by 20 μm for Sholl analysis. Scale bar, 50 µm. ***D*–*R***, Graphs quantifying parameters in 7 month WT and *Gde2*KO CA1 cells (*n* = 16 cells; 4 animals per genotype, mean ± SEM, unpaired *t* test). ***D***, ***E***, Mean somal volume (***D***, *p = 0.0124) and surface area (***E***, *p = 0.0165) of each cell. ***F***, ***G***, Mean length (***F***, ***p = 0.0003) and number of branches (***G***, ns, *p* = 0.0878) for each neuron's apical dendrite. ***H***, ***I***, Average number of total spines (***H***, ns, *p* = 0.8528) and the spine density (***I***, ****p < 0.0001). ***J*–*N***, Spine types along neuron apical dendrites. The mean number of thin spines (***J***, ns, *p* = 0.1009), filopodia spines (***K***, ns, *p* = 0.3619), stubby spines (***L***, ns, *p* = 0.1643), and mushroom spines (***M***, *p = 0.0330), and mushroom spine density as a fraction of the total spine count (***N***, ****p < 0.0001). ***O*–*R***, Sholl analysis of apical dendrites in each concentric circle (see ***B*** and ***C***) for length (***O***, radius = 100 µm, ***p = 0.0003; radius = 120 µm, **p = 0.0066), the number of intersections (***P***, radius = 80 µm, **p = 0.0026; radius = 100 µm, *p = 0.0173; radius = 120 µm, **p = 0.0026), the number of spines (***Q***), and the number of mushroom spines (***R***, radius = 100 µm, ***p = 0.0007; radius = 120 µm, *p = 0.0220). ***O*–*R***, Mean ± SEM, two-way ANOVA. See Extended Data Figure 2-1 for more details. See Table 1 for statistical summaries.

To investigate if GDE2 function in hippocampal CA1 cells, we examined CA1 pyramidal cell synaptodendritic architecture using Golgi–Cox staining in *Gde2*KO mice and compared them with WT animals (Fig. 2). We focused our analysis on animals at 7 months of age because cognitive abnormalities in *Gde2*KO mice begin to emerge at this stage prior to neuronal loss at 19 months (Daudelin et al., 2024; Zhang et al., 2024). After staining hippocampal sections (Fig. 2*A*), we constructed 3D models of individual CA1 pyramidal cells with spine connections using the Neurolucida software (Fig. 2*B*,*C*; see Materials and Methods) to quantify differences in morphology (Fig. 2*D*–*N*) and to evaluate dendritic complexity by Sholl analysis (Fig. 2*O*–*R*). We found that the somas of CA1 pyramidal cells of 7-month-old *Gde2*KOs are significantly enlarged compared with WT controls (Fig. 2*D*,*E*). In addition, *Gde2*KO CA1 pyramidal cells show increased total apical dendrite length compared with WT cells but not an overall increase in the total number of branches (Fig. 2*F*,*G*). Interestingly, Sholl analysis revealed that apical dendrites of *Gde2*KO CA1 pyramidal cells showed increased length and complexity in areas proximal to the cell body compared with WT, with more distal areas showing mostly similar dendritic length and branching between genotypes (Fig. 2*O*,*P*).

10.1523/ENEURO.0102-25.2025.f2-1 Figure 2-1 **GDE2 does not regulate hippocampal synaptic protein amounts in 7-month mice.** (A, C, E) Western blots of 7-month WT and *Gde2*KO hippocampal extracts (n = 4 animals per genotype, unpaired *t* test). Actin is used as a loading control. (A) PSD95 (C) Synaptophysin (E) Homer. (B, D, F) Graphs quantifying western blots normalized to Actin prior to normalizing to WT for (B) PSD95 (ns p = 0**.**2126), (D) Synaptophysin (ns p = 0.6036), and (F) Homer (ns p = 0.1765). (B, D, F) All graphs: mean ± s.e.m.. See Table 1 for statistical summaries. Download Figure 2-1, TIF file.

Analysis of spine numbers in apical dendrites showed no differences in total numbers between *Gde2*KO and WT CA1 pyramidal cells (Fig. 2*H*). In line with this observation, Western blots of 7 month hippocampal lysates showed no changes in the amounts of presynaptic synaptophysin and postsynaptic PSD-95 and Homer proteins between WT and *Gde2*KOs (Extended Data Fig. 2-1). Nevertheless, the spine density of *Gde2*KOs was decreased compared with WT, consistent with longer *Gde2*KO apical dendrite lengths (Fig. 2*I*). Examination of specific spine types along the apical dendrites showed no differences between the number of thin, filopodia, or stubby spines (Fig. 2*J*–*L*). However, both the total number and density of CA1 apical dendrite mushroom spines (as a fraction of total spines) were increased in *Gde2*KO pyramidal cells compared with WT (Fig. 2*M*,*N*). Notably, while total spine numbers were not different throughout the apical dendrites between genotypes (Fig. 2*Q*), the number of mushroom spines in *Gde2*KO pyramidal cells was increased within the portion of the apical dendrite closer to the soma (stratum radiatum) and not the more distal regions (Fig. 2*R*), which overlaps with the regionalized increase of dendritic length and complexity observed in these neurons (Fig. 2*O*–*R*).

Taken together, these observations indicate that the loss of GDE2 alters the dendrite and spine morphology of CA1 hippocampal pyramidal cells. These changes include an increase in dendritic length and complexity and an elevation in the number of mushroom spines, particularly in the stratum radiatum. No changes were detected in other types of spines or in the levels of synaptic proteins, highlighting the specific consequences of GDE2 loss on CA1 cell morphologies.

### GDE2 regulates pre- and postsynaptic neuronal function

**Figure 3.**
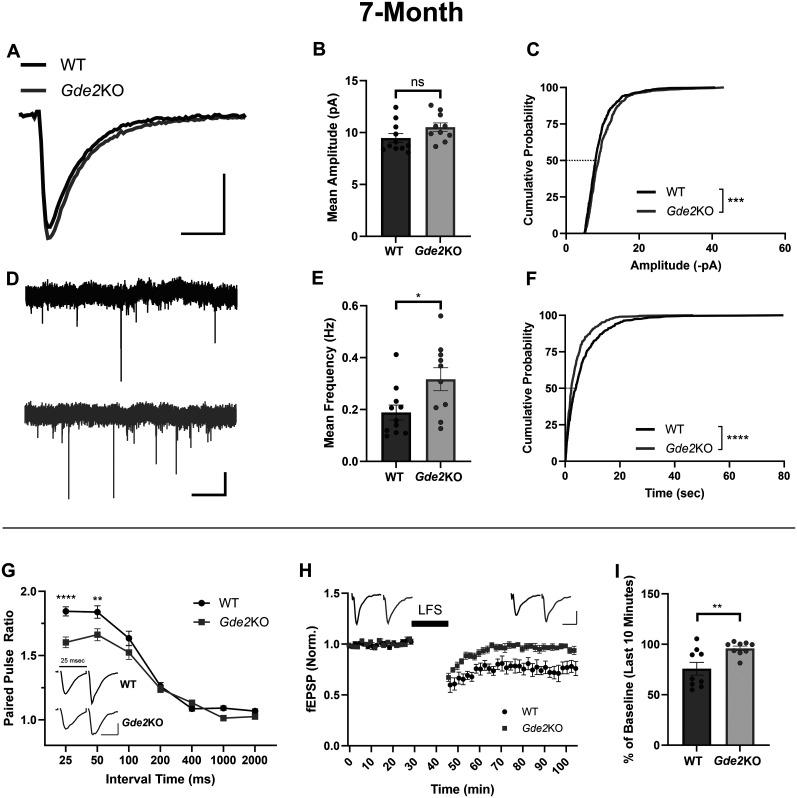
GDE2 impacts pre- and postsynaptic function. ***A–F***, Whole-cell patch–clamp mEPSC recording data from CA1 pyramidal cells of 7-month-old *Gde2*KO and WT animals (*n* = 11 WT cells; *n* = 10 *Gde2*KO cells; 4 animals per genotype). ***A***, Superimposed representative averaged traces aligned by the start of rise time for each genotype (*x*-axis, 5 ms; *y*-axis, 5 pA). ***B***, Graph quantifying the mean amplitude of events for both genotypes (unpaired *t* test, ns, *p* = 0.0976). ***C***, Cumulative distribution of amplitudes, with the dashed line showing 50% cumulative probability (Kolmogorov–Smirnov test, ***p = 0.0009). ***D***, Thirty-second representative raw recording traces (*x*-axis, 5 s; *y*-axis, 10 pA). ***E***, A graph quantifying the mean frequency of events for *Gde2*KO and WT cells (unpaired *t* test, *p = 0.0223). ***F***, Cumulative distribution of event intervals, with the dashed line showing 50% cumulative probability (Kolmogorov–Smirnov test, ****p < 0.0001). ***G*–*I***, Field recordings of 7-month-old *Gde2*KO and WT hippocampal slices in the CA1 region. ***G***, PPF induction of CA1 field excitatory postsynaptic potentials (fEPSPS) in *Gde2*KO and WT slices (*n* = 8 slices from 3 animals per genotype) reported as the ratio of the second fEPSP slope to the first fEPSP slope (*y*-axis) tested using various interstimulus intervals (ISIs) between the first and second pulses (25, 50, 100, 200, 400, 1,000, and 2,000 ms). Representative traces of WT and *Gde2*KO first and second responses at 7 months with 25 ms interval between stimuli (scale bar, *x*-axis, 15 ms; *y*-axis, 0.3 mV). Two-way ANOVA, ISI, 25 ms, ****p < 0.0001; ISI, 50 ms, **p = 0.0034. ***H***, LFS-induced NMDAR-dependent LTD in *Gde2*KO and WT slices at 7 months (*n* = 9 slices, 5 animals per genotype) reported as the ratio of the induced response's fEPSP slope to the average baseline fEPSP slope (first 30 min). Representative traces of WT and *Gde2*KO responses (***H***, top left side) and responses at the end of recording post-NMDAR–dependent LTD induction (***H***, top right side; scale bar, *x*-axis, 15 ms; *y*-axis, 0.3 mV). ***I***, A graph quantifying the average fEPSP slope during the last 10 min of recording data as a percentage of the average fEPSP slope during the baseline for both genotypes (unpaired *t* test, **p = 0.0085). All bar graphs, mean ± SEM. See Extended Data Figures 3-1–3-3 for more details. See Table 1 for statistical summaries.

Given the morphological changes in CA1 pyramidal cells in 7-month-old *Gde2*KO mice, we examined whether CA1 pyramidal cell electrophysiological properties and functions were disrupted in *Gde2*KO animals using whole-cell patch–clamp and field recordings (Fig. 3). Hippocampal slices were prepared from 7-month-old male and female *Gde2*KO and WT animals. CA1 pyramidal cells were patched, and the amplitude and frequency of miniature excitatory postsynaptic currents (mEPSCs) were measured when action potentials were blocked with TTX. Because cumulative probability distribution tests are extremely sensitive and can capture minor, isolated differences between groups (Gordleeva et al., 2023), we considered both mean values of events and cumulative probability distributions when interpreting outcomes. The mean amplitudes of events were unchanged between genotypes (Fig. 3*A*,*B*), with only the cumulative probability distribution showing minor differences between *Gde2*KO and WT mice (Fig. 3*C*). However, there was a significant increase in the frequency of events in *Gde2*KO CA1 pyramidal cells compared with WT neurons, manifested by the decrease in time between events when the mean frequency or the cumulative probability distribution was considered (Fig. 3*D*–*F*). Seven-month-old CA1 pyramidal cells showed no changes in waveform kinetics (Extended Data Fig. 3-1*A*,*B*) or in series and membrane resistances (Extended Data Fig. 3-1*C*,*D*). We further measured the amplitudes and frequency of mEPSCs in CA1 pyramidal cells of juvenile *Gde2*KO and WT animals at 1.5 months. We found no difference between the mean amplitudes (Extended Data Fig. 3-1*E*,*F*) and mean frequencies (Extended Data Fig. 3-1*H*,*I*) of *Gde2*KO and WT events, with differences only detected when the cumulative probability distributions were compared (Extended Data Fig. 3-1*G*–*J*). Similar to the 7 month condition, the kinetics of the mEPSC waveform (Extended Data Fig. 3-1*K*,*L*) along with series and membrane resistances (Extended Data Fig. 3-1*M*,*N*) were unchanged between *Gde2*KO and WT mice at 1.5 months. This is consistent with the expression levels of GDE2 in the hippocampus, which we find to be substantially lower at 1.5 months compared with 7 months of age (Extended Data Fig. 3-2). These observations suggest that the loss of GDE2 function leads to an increase in the frequency of mEPSC events in adult animals, consistent with the potential presynaptic role of GDE2. Moreover, they suggest that GDE2 expressed developmentally does not substantially affect mEPSC properties.

10.1523/ENEURO.0102-25.2025.f3-2 Figure 3-2 **GDE2 loss does not significantly impact mEPSC properties or frequency/amplitudes at 1.5-months.** (A-D) Waveform kinetics and resistance measurements of mEPSCs from 7-month WT and *Gde2*KO CA1 cells (n = 11 WT cells, n = 10 *Gde2*KO cells, 4 animals per genotype, unpaired t-test). Rise time (A, ns p = 0.5239) decay tau (B, ns p = 0.9496), series resistance (C, ns p = 0.6225), and membrane resistances (D, ns p = 0.7059). (E-N) Whole-cell patch-clamp mEPSC recording data from 1.5-month-old *Gde2*KO and WT CA1 pyramidal cells (n = 10 WT cells, n = 11 *Gde2*KO cells, 5 animals per genotype, all bar graphs, unpaired *t*-test). (E) Superimposed representative averaged traces aligned by the start of rise time for each genotype (x-axis: 5 msec, y-axis: 5 pA). (F) Graph quantifying the mean amplitude of events for both genotypes (ns p = 0.0790). (G) Cumulative distribution of amplitudes, with dashed line showing 50% cumulative probability (Kolmogorov-Smirnov test, ****p < 0.0001). (H) 30-second representative raw recording traces (x-axis: 5 sec, y-axis: 10pA). (I) Graph quantifying the mean frequency of events for *Gde2*KO and WT cells (ns p = 0.0949). (J) Cumulative distribution of event intervals, with dashed line showing 50% cumulative probability (Kolmogorov-Smirnov test, ****p < 0.0001). (K-N) Waveform kinetics and resistance measurements of mEPSCs. Rise time (K, ns p = 0.2225), decay tau (L, ns p = 0.9490), series resistance (M, ns p = 0.4825), and membrane resistances (N, ns p = 0.4013). All bar graphs: mean ± s.e.m.. See Table 1 for statistical summaries. Download Figure 3-2, TIF file.

10.1523/ENEURO.0102-25.2025.f3-1 Figure 3-1 **GDE2 hippocampal expression is higher at 7-months compared with 1.5-months.** (A) Representative western blot of hippocampal lysates from 1.5- and 7-month WT mice probing for GDE2 and Actin (n = 4 animals per time point). GDE2 (bracket) typically runs as a series of diffuse bands due to glycosylation; accordingly, *Gde2*KO hippocampal lysate run on the same gel is referenced as a control. The asterisk denotes a non-specific protein band. (B) GDE2 levels are 2.4 fold higher at 7 months compared to 1.5 months after normalization to actin (mean ± s.e.m., unpaired *t* test, p**** < 0.0001). Download Figure 3-1, TIF file.

10.1523/ENEURO.0102-25.2025.f3-3 Figure 3-3 **GDE2 does not impact 1.5-month PPR, NMDAR-dependent LTD, or 7-month mGluR-dependent LTD.** (A) Schematic of CA1 hippocampal slice field recording set up showing stimulation and recording electrode placement in the *stratum radiatum*. (B) Paired-pulse facilitation (PPF) induction of CA1 field EPSPs (fEPSPS) in 1.5-month *Gde2*KO and WT slices (n = 10 WT slices from 5 animals, n = 12 *Gde2*KO cells from 5 animals per genotype) reported as the ratio of the second fEPSP slope to the first fEPSP slope (y-axis) tested using various interstimulus interval (ISI) between the first and second pulses (25, 50, 100, 200, 400, 1000, and 2000 msec). (C and D) LFS-induced NMDAR-dependent LTD in *Gde2*KO and WT slices at 1.5-months (C, n = 7 WT slices, n = 6 *Gde2*KO cells, 4 animals per genotype) reported as the ratio of the induced response’s fEPSP slope to the average baseline fEPSP slope (first 30 minutes) and graph quantifying the average fEPSP slope during the last 10 minutes of recording data as a percent of the average fEPSP slope during baseline for both genotypes (D, ns p = 0.4783, unpaired *t* test). (E and F) DHPG-induced mGluR-dependent LTD in *Gde2*KO and WT slices at 7-months (E, n = 6 WT slices, n = 8 *Gde2*KO cells, 3 animals per genotype) reported as the ratio of the induced response’s fEPSP slope to the average baseline fEPSP slope (first 30 minutes) and graph quantifying the average fEPSP slope during the last 10 minutes of recording data as a percent of the average fEPSP slope during baseline for both genotypes (F, ns p = 0.8145, unpaired *t* test). (D and F) All bar graphs: mean ± s.e.m.. Schematic in A created in BioRender.com. See Table 1 for statistical summaries. Download Figure 3-3, TIF file.

Apical dendrites of CA1 pyramidal cells receive synaptic input from the CA3 region of the hippocampus via the Schaffer collateral pathway (Steward, 1976). We next assessed whether GDE2 regulates short- and long-term hippocampal plasticity within the CA3–CA1 circuit using field recordings (Fig. 3*G*–*I*). We prepared hippocampal slices from male and female 7-month-old *Gde2*KO and WT mice and stimulated adjacent stratum radiatum while recording responses in the CA1 region (Extended Data Fig. 3-3*A*; see Materials and Methods). We investigated short-term synaptic plasticity differences between *Gde2*KO and WT animals by measuring the PPR. The PPR is the ratio of the amplitude of the second postsynaptic response to the first response when two stimuli are delivered in rapid succession, which typically serves as a measure of presynaptic function (Leung and Fu, 1994). We measured PPR at various intervals ranging from 25 to 2,000 ms. At 7 months of age, *Gde2*KO hippocampal slices have significantly reduced PPRs at multiple time intervals (Fig. 3*G*), while at 1.5 months of age, both genotypes exhibit similar PPRs across all time intervals tested (Extended Data Fig. 3-3*B*). These observations suggest that GDE2 expression in adult mice is required for short-term hippocampal plasticity and is consistent with proposed presynaptic localization and functions for GDE2.

Previous behavioral studies in *Gde2*KO animals identified deficits in fear-conditioned memory associated with NMDAR-LTD, while no changes were observed in fear memory extinction, in which metabotropic glutamate receptor (mGluR)-LTD is implicated (Zhu et al., 2017; Daudelin et al., 2024). Accordingly, we examined long-term synaptic plasticity in *Gde2*KO animals by inducing NMDAR-dependent LTD using an LFS protocol (see Materials and Methods). This paradigm, which produces a decrease in postsynaptic strength, would also uncover potential postsynaptic functions for GDE2. At 7 months, *Gde2*KO mice showed significantly impaired NMDAR-dependent LTD compared with WT animals (Fig. 3*H*,*I*), while at 1.5 months, NMDAR-dependent LTD was equivalent between genotypes (Extended Data Fig. 3-3*C*,*D*). Consistent with previous behavioral studies (Daudelin et al., 2024), dihydroxyphenylglycine-induced mGluR–dependent LTD showed no differences between genotypes (Extended Data Fig. 3-3*E*,*F*).

Collectively, these observations suggest that GDE2 expression in adult mice is required for both pre- and postsynaptic function at Schaffer collateral→CA1 synapses, including short- and long-term plasticity.

### GDE2 regulates hippocampal AKT-GSK3 signaling

**Figure 4.**
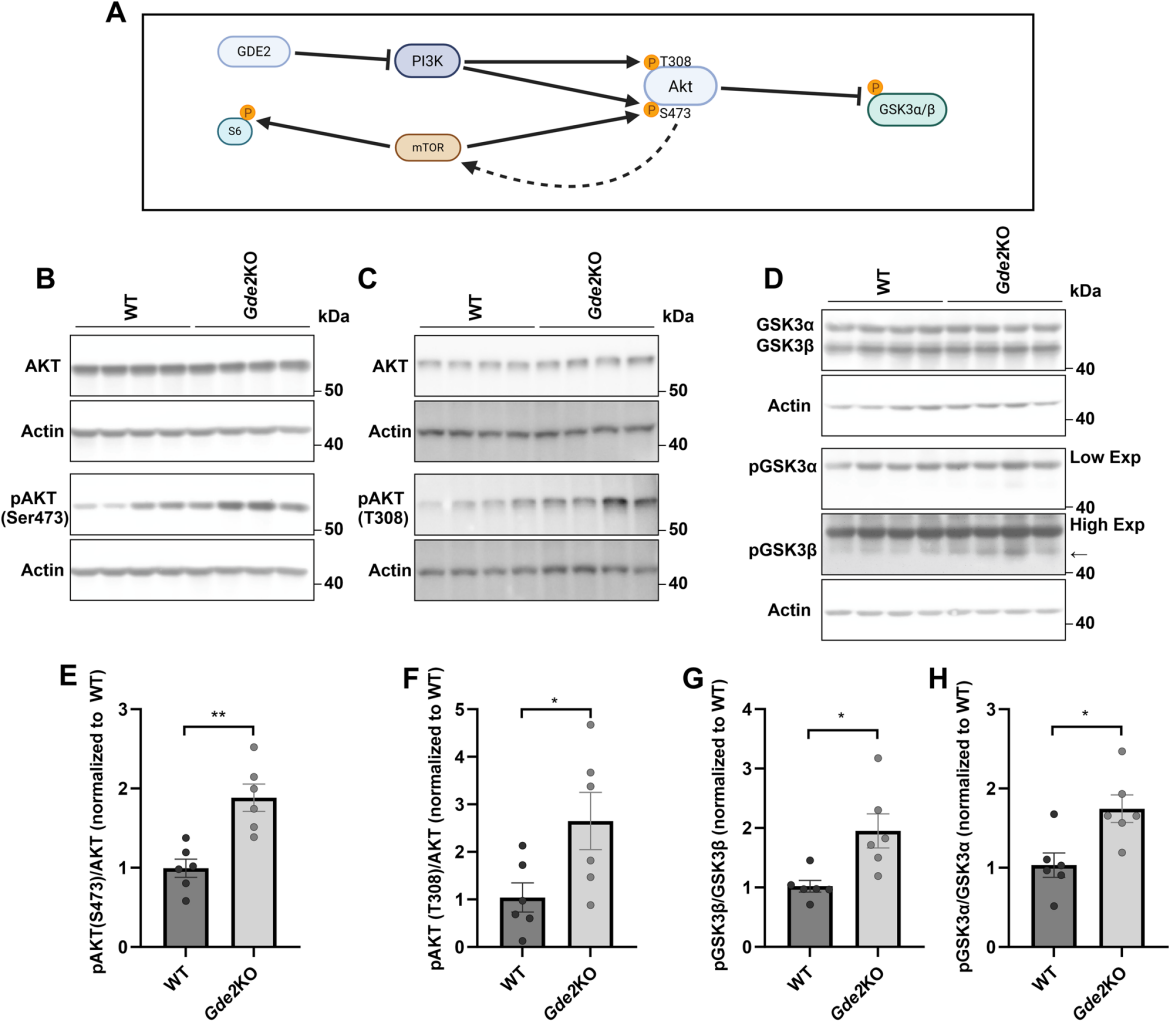
The PI3K-AKT-GSK3 signaling pathway is regulated by GDE2. ***A***, Schematic showing that PI3K phosphorylates and activates Akt, which subsequently phosphorylates and inhibits GSK activity. Akt and S6 kinase are also substrates of mTOR, with a potential feedback loop that promotes mTOR activity (dotted arrow). GDE2 is hypothesized to act at the cell surface to negatively regulate PI3K activity and downstream Akt-dependent inhibition of GSK. ***B*–*D***, Western blots of 7 month WT and *Gde2*KO hippocampal extracts (*n* = 6 animals per genotype). Actin is used as a loading control. ***B***, Total and phosphorylated AKT (S473; ***C***) total and phosphorylated AKT (T308), (***D***) total and phosphorylated GSK3β and GSK3ɑ (high and low exposures for phosphorylated GSK3 blots are shown). The arrow highlights pGSK3β. ***E***, ***F***, Graphs quantifying Western blots normalized to Actin prior to normalizing to WT. ***E***, The ratio of phosphorylated AKT (S473) to total AKT (**p = 0.0016). ***F***, The ratio of phosphorylated AKT (T308) to total AKT (*p = 0.0386). ***G***, ***H***, The ratio of phosphorylated GSK3β (***G***, *p = 0.0119) and GSK3ɑ (***H***, *p = 0.0120) to total hippocampal GSK3β and GSK3ɑ, respectively. All graphs: mean ± SEM, unpaired *t* test. See Extended Data Figures 4-1 and 4-2 for more details. Schematic in ***A*** is created in BioRender.com. See Table 1 for statistical summaries.

Previous studies have shown that the phosphoinositide 3-kinase-protein kinase B (PI3K-AKT) pathway inhibits glycogen synthase kinase β (GSK3β) activity to block the induction of NMDAR-mediated LTD (Peineau et al., 2007). Mechanistically, stimulation of PI3K leads to activation of AKT through phosphorylation at Serine (S) 473 and Threonine (T) 308 (Fig. 4*A*; Glaviano et al., 2023). Activated AKT, in turn, inhibits GSK3β activity by phosphorylation at S9 (Fig. 4*A*), which prevents NMDAR-mediated LTD (Peineau et al., 2007). Accordingly, we examined if the deregulation of the PI3K-AKT-GSK3β signaling axis underlies the reduced hippocampal NMDAR-mediated LTD induction in adult *Gde2*KO mice. We harvested hippocampal tissue from 7-month-old *Gde2*KO and WT mice and assessed total and phosphorylated AKT levels via Western blot (Fig. 4). Total amounts of AKT remained unchanged between genotypes (Fig. 4*B*; Extended Data Fig. 4-1*A*); however, *Gde2*KO mice showed increased levels of phosphorylated AKT at S473 (Fig. 4*B*,*E*). Both PI3K and the mammalian target of rapamycin (mTOR) can phosphorylate AKT at S473 (Fig. 4*A*; Glaviano et al., 2023). However, total and phosphorylated amounts of S6 kinase, a downstream target of mTOR, were unchanged between genotypes (Extended Data Fig. 4-1*E*–*G*). Moreover, the amount of phosphorylated AKT at T308, a second site of PI3K phosphorylation on AKT, was increased in *Gde2*KOs compared with WT (Fig. 4*C*,*F*; Extended Data Fig. 4-1*B*). These observations suggest that PI3K-dependent activation of AKT is specifically increased in the *Gde2*KO hippocampus.

10.1523/ENEURO.0102-25.2025.f4-1 Figure 4-1 **GDE2 does not regulate total AKT, GSK3, S6, and phosphorylated S6 in 7-month hippocampus.** (A-D) Graphs quantifying western blots from 7-month WT and *Gde2*KO hippocampal extracts (n = 6 animals per genotype, unpaired *t* test) normalized to Actin prior to normalizing to WT. See main Figure 4 for blots. (A and B) AKT (AKT (S473) blot: ns p = 0.5782, AKT (T308) blot: ns p = 0.6481), (C) GSK3β (ns p = 0.5931), and (D) GSK3ɑ (ns p = 0.2083). (E) Western blots of 7-month WT and *Gde2*KO hippocampal extracts (n = 4 animals per genotype) for S6 and phosphorylated S6. Actin is used as a loading control. (F and G) Graphs quantifying western blots normalized to Actin prior to normalizing to WT for (F) S6 (ns p = 0.3130) and (G) phosphorylated S6 (ns p = 0.3850), unpaired *t* test. All graphs: mean ± s.e.m.. See Table 1 for statistical summaries. Download Figure 4-1, TIF file.

10.1523/ENEURO.0102-25.2025.f4-2 Figure 4-2 **GDE2 does not modulate hippocampal phosphorylated AKT and GSK3 in 1.5-month mice.** (A, D) Western blots of 1.5-month WT and *Gde2*KO hippocampal extracts (n = 4 animals per genotype). Actin is used as a loading control. (A) total and phosphorylated AKT (S473), (D) total and phosphorylated GSK3β and GSK3ɑ. (B, C, E-H) Graphs quantifying western blots normalized to Actin prior to normalizing to WT for (B) total AKT (**p = 0.0033), (C) phosphorylated AKT (S473) to total AKT (ns p = 0.2173), (E) total GSK3ɑ (ns p = 0.1375), (F) phosphorylated GSK3ɑ to total GSK3ɑ (ns p = 0.3509), (G) total GSK3β (ns p = 0.1155), (H) phosphorylated GSK3β to total GSK3β (ns p = 0.3224). All graphs: mean ± s.e.m., unpaired *t* test. See Table 1 for statistical summaries. Download Figure 4-2, TIF file.

We next examined the phosphorylation of GSK3β by Western blot of hippocampal lysates prepared from 7-month-old *Gde2*KO and WT mice. Total amounts of GSK3β were equivalent across genotypes (Fig. 4*D*; Extended Data Fig. 4-1*C*); however, the amount of phosphorylated GSK3β was significantly increased in *Gde2*KO hippocampal lysates (Fig. 4*D*,*G*). Of note, GSK3α, related to GSK3β, is also implicated in regulating NMDAR-mediated LTD, and its activity is inhibited through phosphorylation at S21 by activated AKT (Draffin et al., 2021). Interestingly, the amounts of phosphorylated GSK3α (S21) are also elevated in the *Gde2*KO hippocampus in adult mice, while total levels of GSK3α are not changed (Fig. 4*D*,*H*; Extended Data Fig. 4-1*D*). Taken together, these observations suggest that hippocampal GSK3 isoforms are inhibited in adult mice lacking GDE2 via the activation of the PI3K-AKT signaling pathway, supporting the model that the disruption of the PI3K-AKT-GSK3 signaling axis mediates the NMDAR-LTD in *Gde2*KO animals. In line with this model, we detected no significant differences in the proportion of phosphorylated AKT, GSK3α, and GSK3β between WT and *Gde2*KO mice at 1.5 months of age, a time when no deficits in NMDAR-mediated LTD are detected in *Gde2*KO animals (Extended Data Fig. 4-2).

### PI3K inhibition rescues NMDAR-LTD deficit in *Gde2*KO mice

**Figure 5.**
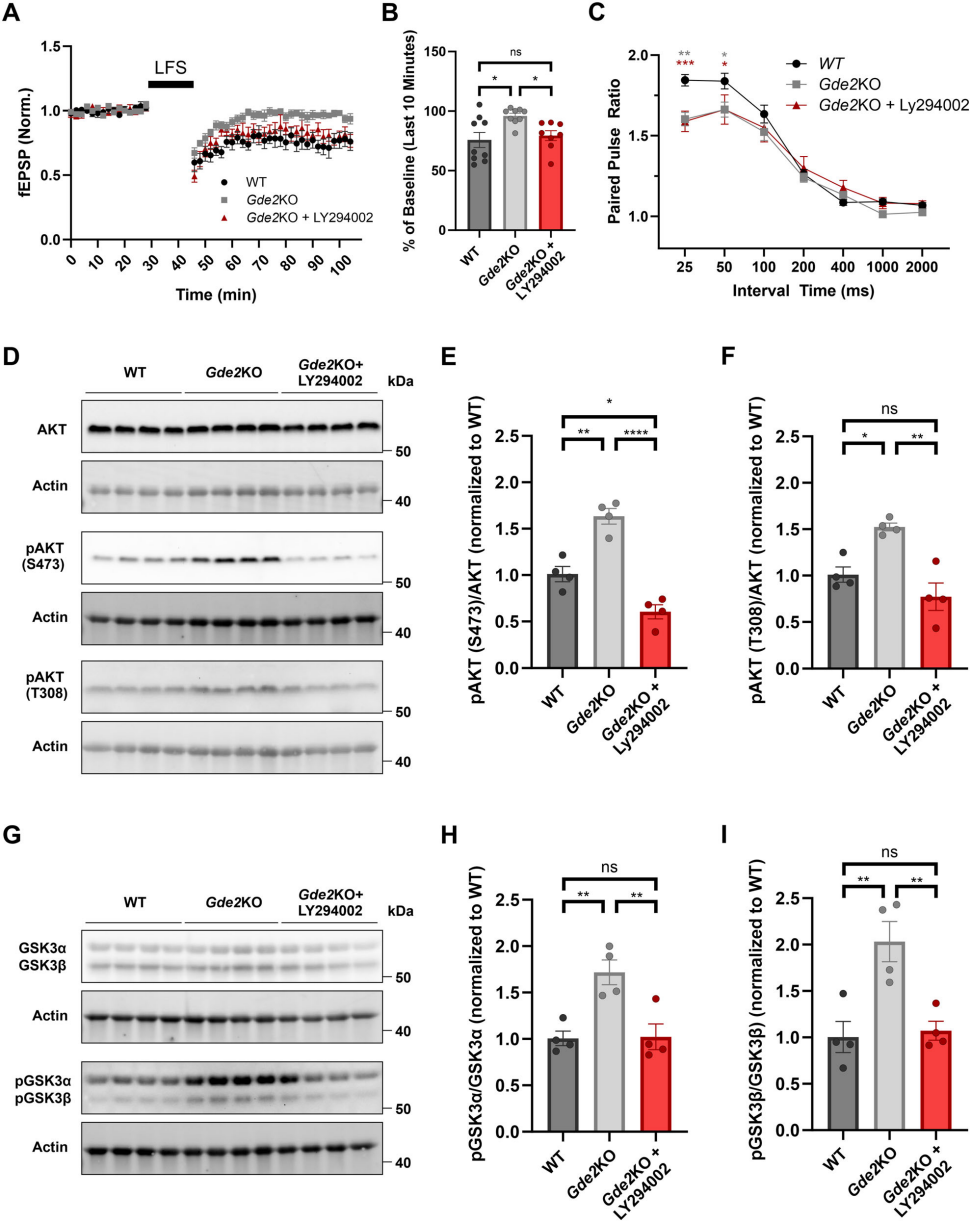
PI3K inhibition rescues NMDAR-dependent LTD deficits in *Gde2*KO hippocampal slices. ***A***, LFS-induced NMDAR–dependent LTD in slices from 7-month-old WT, *Gde2*KO, and *Gde2*KO with the PI3K inhibitor LY294002 applied, reported as the ratio of the induced response's fEPSP slope to the average baseline fEPSP slope (first 30 min, WT and *Gde2*KO data from Fig. 3, *n* = 8 slices from 6 animals for *Gde2*KO + LY294002 condition). ***B***, A graph quantifying the average fEPSP slope during the last 10 min of recording data as a percentage of the average fEPSP slope during baseline for all conditions (WT and *Gde2*KO data from Fig. 3, one-way ANOVA, WT vs *Gde2*KO, *p = 0.0118; *Gde2*KO vs *Gde2*KO + LY294002, *p = 0.0496; WT vs *Gde2*KO + LY294002, ns, *p* = 0.8445). ***C***, PPF induction of CA1 fEPSPs in WT, *Gde2*KO, and *Gde2*KO slices + LY294002 (WT and *Gde2*KO data from Fig. 3; *n* = 8 slices; 4 animals for *Gde2*KO + LY294002 condition) reported as the ratio of the second fEPSP slope to the first fEPSP slope (*y*-axis) tested using the same ISIs in Figure 3 (two-way ANOVA, ISI, 25 ms, WT vs *Gde2*KO, **p = 0.001; WT vs *Gde2*KO + LY294002, ***p = 0.0005; ISI, 50 ms, WT vs *Gde2*KO, *p = 0.223; WT vs *Gde2*KO + LY294002, *p = 0.0229). ***D***, ***G***, Western blots of 7 month hippocampus slice extracts (WT, *Gde2*KO, and *Gde2*KO slices treated with LY294002) prepared and incubated in ACSF (*n* = 4 animals per condition). Actin is used as a loading control. ***D***, Total and phosphorylated AKT (S473 and T308). ***G***, Total and phosphorylated hippocampal GSK3ɑ and GSK3β. ***E***, ***F***, Graphs quantifying the ratio of phosphorylated AKT to total AKT normalized to Actin and then normalized to WT levels for AKT (S473) (***E***, one-way ANOVA, WT vs *Gde2*KO, **p = 0.0011; *Gde2*KO vs *Gde2*KO + LY294002, ****p < 0.0001; WT vs *Gde2*KO + LY294002, *p = 0.0156) and AKT (T308; ***F***, one-way ANOVA, WT vs *Gde2*KO, *p = 0.0140; *Gde2*KO vs *Gde2*KO + LY294002, **p < 0.0013; WT vs *Gde2*KO + LY294002, ns, *p* = 0.2668). ***H***, ***I***, Graphs quantifying the ratio of phosphorylated GSK3 proteins to total GSK3 normalized to Actin and then normalized to WT levels for pGSK3α (***H***, one-way ANOVA, WT vs *Gde2*KO, **p = 0.0059; *Gde2*KO vs *Gde2*KO + LY294002, **p = 0.0068; WT vs *Gde2*KO + LY294002, ns, *p* = 0.9942) and pGSK3β (***I***, one-way ANOVA, WT vs *Gde2*KO, **p = 0.0051; *Gde2*KO vs *Gde2*KO + LY294002, **p = 0.0077; WT vs *Gde2*KO + LY294002, ns, *p* = 0.9558). All bar graphs, mean ± SEM. See Extended Data Figure 5-1 for more details. See Table 1 for statistical summaries.

Our model posits that PI3K is abnormally activated in the *Gde2*KO hippocampus and that PI3K-mediated activation of AKT and subsequent inhibition of GSK3 activity underlies the NMDAR-mediated LTD deficits in *Gde2*KO mice. If this is the case, then inhibition of PI3K should rescue the NMDAR-mediated LTD loss in *Gde2*KOs. LY294002 is a commonly used broad inhibitor of PI3K that acts as a competitive inhibitor for ATP binding in the active site of PI3K (Gharbi et al., 2007). We incubated hippocampal slices prepared from 7-month-old WT and *Gde2*KO animals in ACSF or ACSF with LY294002 for 2 h prior to implementing the LFS-induced LTD protocol (see Materials and Methods). While incubation with LY294002 did not influence NMDAR-mediated LTD in WT animals (Extended Data Fig. 5-1*A*,*B*), LY294002 treatment rescued the LFS-induced LTD in *Gde2*KO slices to WT levels (Fig. 5*A*,*B*). Interestingly, the application of LY294002 failed to rescue the PPR short-term plasticity deficits seen in 7-month-old *Gde2*KO slices (Fig. 5*C*), suggesting the involvement of other pathways regulated by GDE2 in this process.

10.1523/ENEURO.0102-25.2025.f5-1 Figure 5-1 LY294002 does not reduce WT NDMAR-dependent LTD or affect total AKT and GSK3 levels in *Gde2*KO slices (A) LFS-induced NMDAR-dependent LTD in slices from 7-month WT and WT with LY294002 applied, reported as the ratio of the induced response’s fEPSP slope to the average baseline fEPSP slope (first 30 minutes, WT data from Fig. 3, n = 7 slices from 5 animals for WT + LY294002 condition). (B) Graph quantifying the average fEPSP slope during the last 10 minutes of recording data as a percentage of the average fEPSP slope during baseline (WT data from Fig. 3, unpaired *t* test, ns p = 0.7193). (C – E) Graphs quantifying western blots from 7-month WT, *Gde2*KO, and *Gde2*KO slices treated with LY294002 (n = 4 animals per condition) normalized to Actin prior to normalizing to WT for total AKT (C, one-way ANOVA, ns p > 0.05), GSK3α (D, one-way ANOVA, ns p > 0.05) and pGSK3β (E, one-way ANOVA, ns p > 0.05). All bar graphs: mean ± s.e.m.. See Table 1 for statistical summaries. Download Figure 5-1, TIF file.

LY294002 is an established inhibitor of PI3K, but it also inhibits other kinases such as CK2, PIM1, mTOR, and PLK1 (Gharbi et al., 2007), raising the possibility that LY294002 treatment of *Gde2*KO slices may affect other kinases besides PI3K. CK2 and PLK1 do not phosphorylate both AKT S473, AKT T308, and GSK3 isoforms, while mTOR signaling is not disrupted in the *Gde2*KO hippocampus (Extended Data Fig. 4-1*E*–*G*; Di Maira et al., 2009; Xu et al., 2010), thus ruling out involvement of these kinases in GDE2-dependent regulation of AKT/GSK signaling. PIM1 does not phosphorylate AKT but is known to phosphorylate GSK3 directly (Warfel and Kraft, 2015; Chauhan et al., 2024). Thus, it is possible that the rescue of NMDAR-mediated LTD in *Gde2*KO slices by LY294002 is mediated by the inhibition of PIM1 and not through PI3K. To address this question, we prepared hippocampal slices from 7-month-old WT and *Gde2*KOs and incubated slices in ACSF or LY294002 for 2 h as per our electrophysiology experiments. Western blot analysis of extracts prepared from the slices showed increased phosphorylated AKT (S473 and T308) and phosphorylated GSK3 isoforms (S9/S21) in *Gde2*KO slices compared with WT (Fig. 5*D*–*I*). Notably, LY294002 treatment significantly decreased both phosphorylated AKT and GSK3 isoforms to WT levels, except for phosphorylated AKT S473, which was decreased even further, while leaving total levels of AKT and GSK3 unchanged (Fig. 5*D*–*I*; Extended Data Fig. 5-1*C*–*E*). These observations suggest that GDE2 acts through the PI3K-AKT-GSK3 pathway to modulate NMDAR-mediated LTD.

## Discussion

Synaptic activity and plasticity are tightly regulated via a complex network of pathways that, when misregulated, can result in cognitive impairment (Lepeta et al., 2016). Here, we show that the six-transmembrane surface GPI-anchor cleaving enzyme GDE2 is required for regulating key signaling pathways important for appropriate hippocampal CA1 cellular morphology and synaptic function. GDE2 is localized throughout hippocampal apical dendrites and is present at pre- and postsynaptic sites. Adult mice lacking GDE2 showed morphological changes in CA1 cells, including somal enlargement and increases in apical dendritic lengths, branching of apical dendrites, and the number of mushroom spines. These changes were accompanied by functional alterations in hippocampal synaptic activity, evidenced by pre- and postsynaptic differences in *Gde2*KO mice, including elevated frequency of mEPSCs, reduced PPR, and diminished NMDAR-mediated LTD. *Gde2*KO hippocampal extracts showed enhanced activation of the PI3K-AKT-GSK3 signaling pathway and pharmacological inhibition of PI3K by LY294002 restored phosphorylated AKT and GSK3 and NMDAR-mediated LTD to WT levels. These observations highlight the pivotal role of GDE2 in regulating hippocampal synaptic function and plasticity, in part through modulation of the PI3K-AKT-GSK3 signaling cascade.

GDE2 expression in the hippocampus is temporally regulated with minimal expression in juvenile animals (1.5 months) and substantially higher expression at 7 months, the time when we observed multiple phenotypes in *Gde2*KO animals. GDE2 loss affects multiple aspects of hippocampal CA1 cell morphology, which may have consequences for synaptic function and cognitive behavior. The increased somal size of CA1 hippocampal cells in rat models of vascular dementia is associated with cognitive deficits, evidenced by memory impairments in the Morris water maze task (Langdon et al., 2013). In addition, CA1 somal hypertrophy is observed in patients with asymptomatic AD, and neuronal hypertrophy is evident in cortical areas of patients with mild cognitive impairment, consistent with the hypothesis that the increased somal size presages cognitive dysfunction (Iacono et al., 2008). These observations are interesting, considering that *Gde2*KO animals display CA1 somal enlargement and memory deficits when tested in the Morris water maze and that GDE2 distribution and function are disrupted in AD (Nakamura et al., 2021; Daudelin et al., 2024). It is possible that the increased somal size in *Gde2*KOs may be due to impaired neuronal health and viability. While we cannot rule this out, several observations suggest that at 7 months, *Gde2*KO hippocampal neuronal health is unlikely to be impaired. For example, *Gde2*KO CA1 cells are equivalent to WT in terms of total spine numbers, total synaptic proteins, numbers of thin, filopodia and stubby spines, and neuronal properties such as waveform kinetics, series, and membrane resistances. Furthermore, rescue of NMDAR-LTD deficits in *Gde2*KO slices occurs within 3 h of treatment with PI3K inhibitors, indicating that there are no major structural/cellular abnormalities that impact synaptic function. Notably, neuronal loss in *Gde2*KOs emerges at 19 months of age suggesting that GDE2 function in neuronal survival occurs at later stages in life (Zhang et al., 2024). Other morphological changes observed in *Gde2*KO animals include increased CA1 apical dendritic length, complexity, and mushroom spine numbers. Strikingly, these structural changes are all specifically localized to the stratum radiatum, which receives inputs primarily from CA3 cells via the Schaffer collaterals and, in principle, could mediate the altered properties and synaptic activity of this circuit in *Gde2*KO animals (discussed below; Steward, 1976). The mechanisms that cause morphological changes in *Gde2*KO CA1 cells and their spatial restriction remain unknown. It is notable that activated PI3K and AKT are increased in the *Gde2*KO hippocampal tissue when these changes in CA1 cells are observed and that PI3K and AKT activation are implicated in the increasing hippocampal CA1/CA3 cell body size, dendritic length, complexity, and mushroom spine numbers (Kumar et al., 2005). These associations suggest that GDE2 regulation of PI3K-AKT signaling may be involved in regulating the spatially localized morphologies of CA1 hippocampal cells; however, this possibility requires further investigation.

Our observations that GDE2 is localized in pre- and postsynaptic compartments, combined with the localized hippocampal cellular morphological differences in the stratum radiatum of *Gde2*KO CA1 cells, led us to investigate whether *Gde2*KO animals exhibit changes in synaptic function using whole-cell patch–clamp and field recordings. No major changes in synaptic activity were detected in 1.5-month-old *Gde2*KO animals compared with WT, consistent with the low level of GDE2 expression in the hippocampus at this time point as assayed by Western blot. However, we detected several changes in adult *Gde2*KO animals at 7 months, when GDE2 hippocampal expression is substantially elevated. Whole-cell recordings in 7 month *Gde2*KO hippocampal slices revealed increased mEPSC frequency, which has been associated with increased numbers of mushroom spines, a phenotype observed in *Gde2*KO CA1 cells (Sala et al., 2001). Furthermore, field recordings involving the CA3/CA1 Schaffer collateral circuit showed decreased PPR and NMDAR-LTD, consistent with GDE2 localization in pre- and postsynaptic compartments. Our findings that GDE2 loss increases mEPSC frequency and decreases PPR suggests the possibility that the loss of GDE2 enhances vesicle fusion to increase the probability of vesicle release. It will be interesting to determine if this is the case and to define the mechanisms involved. Interestingly, we found that GDE2 ablation does not broadly affect LTD, as mGluR-LTD is unchanged in *Gde2*KO animals, underscoring the specificity of GDE2 function. These observations are consistent with behavioral studies in *Gde2*KO animals that show deficits in fear-conditioned memory associated with NMDAR-LTD, but not fear memory extinction, in which mGluR-LTD is implicated (Liu et al., 2014; Zhu et al., 2017; Daudelin et al., 2024). Previous studies have identified another type of synaptic plasticity, long-term potentiation (LTP), as an inhibitor of NMDAR-LTD (Peineau et al., 2007). Whether LTP is also affected in *Gde2*KO mice is an open question and awaits further investigation.

Our biochemical studies suggest a molecular explanation for the selective function of GDE2 in NMDAR-LTD. Previous work has identified critical roles of GSK3β in specifically regulating NMDAR-LTD and not mGluR-LTD downstream of the PI3K-AKT pathway (Peineau et al., 2007). Interestingly, we find that the PI3K-AKT-GSK3 signaling axis is activated in the *Gde2*KO hippocampus at 7 months of age, resulting in increased levels of inactive pGSK3. Furthermore, inhibition of PI3K activity by treatment with the PI3K inhibitor LY294002 led to reduced active pAKT, the concomitant reduction of inactive pGSK3, and the rescue of NMDAR-LTD. These observations identify GDE2 as a novel regulator of the PI3K-AKT-GSK3 signaling axis important for hippocampal synaptic function and plasticity. While our results indicate that PI3K inhibition rescues deficits in NMDAR-LTD in *Gde2*KO mice, no changes were observed in PPRs when phosphorylated AKT and GSK3α/β levels were reduced via PI3K inhibition. These findings suggest that GDE2 influences hippocampal synaptic function through multiple signaling pathways. Further studies will be essential to fully elucidate the complex signaling networks involved.

GDE2 acts on the cell surface to cleave the GPI anchor that tethers select proteins to the cell membrane (Rao and Sockanathan, 2005; Yanaka, 2007; Park et al., 2013). Accordingly, we hypothesize that GDE2 regulates synaptic function and plasticity through the cleavage of relevant GPI-anchored proteins. Many GPI-anchored proteins are known to be important for synapse development and activity, including NgRs, contactins, RTN4Rs, glypican (GPC) 4, and GPC6, among others (Shimoda and Watanabe, 2009; Allen et al., 2012; Wills et al., 2012; Farhy-Tselnicker et al., 2017; Wang et al., 2021). GPC4 and GPC6, which are members of the GPC family of GPI-anchored heparan sulfate proteoglycans, are established substrates of GDE2 and are required for synapse development and function (Allen et al., 2012; Park et al., 2013; Matas-Rico et al., 2016; Farhy-Tselnicker et al., 2017). In addition, studies in adipocytes show that GPC4 can promote the PI3K-dependent activation of AKT (Ussar et al., 2012). Whether GDE2 regulates GPC4 surface expression and activity to regulate the PI3K-AKT-GSK3 pathway in NMDAR-LTD remains to be elucidated. Nonetheless, a deeper understanding of the mechanism of GDE2 function will provide new insight into the molecular pathways that regulate hippocampal synaptic activity and plasticity.

In summary, we have identified GDE2 as having roles in regulating the morphology and synaptic function of CA1 cells in the adult hippocampus and shown that it contributes to the regulation of the PI3K-AKT-GSK3 signaling pathway involved in mediating NMDAR-LTD. These findings provide insight into the fundamental processes regulating synaptic activity and plasticity in the adult nervous system and may shed light on pathways that are dysregulated in human pathologies that affect synaptic function, memory, and cognition.

## Synthesis

Reviewing Editor: Mark Sheffield, University of Chicago Division of the Biological Sciences Decisions are customarily a result of the Reviewing Editor and the peer reviewers coming together and discussing their recommendations until a consensus is reached. When revisions are invited, a fact-based synthesis statement explaining their decision and outlining what is needed to prepare a revision will be listed below. The following reviewer(s) agreed to reveal their identity: Shuwen Chang, Huaye Zhang.

The manuscript investigates the role of GDE2 in regulating synaptic plasticity and neuronal morphology in the hippocampal CA1 region across two developmental stages. Both reviewers acknowledge the study's potential to provide new insights into the function of GDE2 in synaptic plasticity. However, they raise significant concerns that need to be addressed.

A primary issue is the age dependency of the observed effects, as all phenotypes are present at 7 months but not at 1.5 months. This raises the possibility that the changes seen at 7 months are secondary to GDE2's role in neuronal survival rather than direct effects on synaptic function. Further investigation is needed to determine whether the phenotypes result from age-related compensatory mechanisms rather than GDE2-specific functions.

Additionally, the synaptic localization of GDE2 needs clearer validation, including a quantitative analysis of colocalization with presynaptic and postsynaptic markers. The methodology for identifying GDE2's localization at synapses should be strengthened to support the claims made. The reviewers also recommend a more thorough statistical analysis, including normality testing and improved interpretation of cumulative distribution results.

Both reviewers agree that the manuscript requires substantial revisions to address these concerns, particularly regarding the interpretation of developmental differences and the specificity of GDE2's role in synaptic regulation.

The manuscript has potential, but additional experiments and methodological clarifications are needed to substantiate the conclusions. Detailed reviewer comments follow below.

Reviewer 1:

The authors used different methods to examine the subcellular localization of GDE2 at presynaptic and postsynaptic sites. This may be due to limitations of the GDE2 antibody in accurately reporting its location. Nevertheless, the results indicate that GDE2 is expressed along dendrites but is not highly localized at synapses. In Fig. 1F, GDE2 appears to colocalize more strongly with the presynaptic marker Bassoon than with the postsynaptic marker PSD-95. Could the authors provide a quantitative analysis of the colocalization coefficient of GDE2 with Bassoon and PSD-95 in cultured neurons? Additionally, in Fig. 1H, the PSD-95 staining appears highly noisy and close to background levels, raising concerns about its reliability. Regarding the use of Mander's coefficient (0-1) to assess colocalization, does a value of 0.1 truly indicate colocalization? Could the authors provide a more detailed explanation of how the colocalization ratio was calculated?

The phenotype of GDE2 knockout was observed at 7 months but not at 1.5 months, and it is unclear whether this is due to a developmental increase in GDE2 expression. In line 393, the authors mention that GDE2 is expressed developmentally, but no supporting data is provided. Would it be possible to examine the developmental changes in GDE2 expression in WT animals using western blot analysis? This would help clarify the importance of GDE2 at later developmental stages.

While the authors propose a potential mechanism for GDE2 function as an enzyme that removes GPI, its direct downstream targets remain unclear. In Figure 4, it is difficult to understand how GDE2 fits into the signaling pathway. Could the authors add GDE2's position in the diagram and provide a more detailed description of its role in PI3K signaling to improve clarity?

The finding that GDE2 knockout increases mEPSC frequency and reduces paired-pulse facilitation suggests that GDE2 loss enhances vesicle fusion and increases the probability of vesicle release. While the authors attribute this effect to an increased number of synapses (Lines 544-545), it is also possible that GDE2 knockout directly promotes vesicle fusion. The presynaptic effects of GDE2 were not discussed in detail. It would be beneficial to include a discussion of potential presynaptic targets of GDE2.

Minor Points:

Line 181: The specific confocal imaging equipment is not mentioned-only "Airyscan" and "Zen Blue" are provided. It would be helpful to specify the exact confocal microscope used.

Line 302: A normality test is required to determine the appropriate statistical test. It is unclear from the methods whether the authors tested the data distribution.

Line 318: The full names of neuronal markers (NPTXR, GluN1, GluA1) should be provided for better clarity.

Line 337: "Airyscan" is mentioned without sufficient context, which may be unclear to readers unfamiliar with the term.

Lines 480-483: The rationale behind testing the hypothesis is unclear, as the experimental data already support GDE2's involvement in PI3K-AKT-GSK3 signaling. Perhaps the authors intend to rule out off-target effects of LY294002 on other kinases, such as CK2 or PIM1. If so, they should rephrase this section for clarity.

Lines 551-552: The decision to measure LTD instead of LTP is not well explained in the results section, even though it is discussed later. To improve logical flow, the rationale should be introduced in the results section rather than in the discussion.

Figure 1G: "Uninjected" should be corrected to "non-injected."

Figures 2 and 4: The sequence of different panels does not follow a clear left-to-right, top-to-bottom order. Consider reorganizing them for better readability.

Reviewer 2:

In this manuscript, the authors explored the role of the GPI-anchor cleaving enzyme, GDE2, in hippocampal synaptic morphology and function. They found Gde2KO CA1 cells showed increased dendritic length and complexity and increased numbers of mushroom spines. Further, adult Gde2KOs displayed an increased mEPSC frequency, impaired paired-pulse facilitation (PPF), and disrupted NMDAR-mediated LTD. They also found increased PI3K-AKT-GSK3 signaling in Gde2KO hippocampus, and inhibition of PI3K restored Gde2KO NMDAR-mediated LTD.

While the data are potentially interesting and overall rigorously performed, one major caveat is that all their observed phenotypes are seen at 7 months but not 1.5 months. If the GDE2 plays a role in synaptic function, the effects should be detectable at 1.5 months. Since GDE2 is known to be important for adult neuron survival, this raises the concern that their observed effects are secondary to the role of GDE2 on survival. The authors need to determine whether their observed phenotypes are secondary to the effects of GDE2 loss on survival. If not, they need to determine why the synaptic phenotypes are age-dependent. Specific points are listed below:

1. The authors conclude from Figure 1 that GDE2 is enriched at the pre- and post-synapse. However the data supporting this conclusion are not convincing. The synaptosome data only show the presence of GDE2 but not enrichment. The immunostaining data show GDE2 exhibiting punctate localization pattern. While the authors point to a few of the puncta that do colocalize with Bassoon or PSD-95, overall the GDE2 puncta do not seem to colocalize very well with these synaptic markers. The authors should include a control with randomized puncta to determine whether the colocalization of GDE2 with synaptic markers exceeds chance levels.

2. In Figure 2, the authors used Golgi staining to examine dendritic branching in 7 months old Gde2KO mice. They stated that they focused on this age because that is when cognitive deficits begin to emerge in these mice. However, if GDE2 plays a role in dendritic branching, it should be a developmental effect and should show up much earlier. Since previous studies show GDE2 is important for neuronal survival in adults, examining dendritic complexity at 7 months raises the concern that the effects are secondary to its role in the survival pathways rather than a direct role on dendritic branching. In addition, the fact that they observe increased soma size also suggest this may be an adaptive response to increased stress due to effects on survival pathways, as neurons often show enlarged soma under stress conditions.

3. In Figure 2, representative images of the dendritic spines from WT and Gde2KO neurons should be shown.

4. In Figure 3B and C, the authors show no statistical differences in the mean amplitude of mEPSCs between 7 months old WT and Gde2KO neurons. However, they show a statistical different in the cumulative probability of the amplitude distribution using the Kolmogorov-Smirnov (KS) test. However they did not discuss the significance of this finding. The KS test detects differences in the cumulative distribution function. It is unclear why the WT and KO neurons will show a difference in the sample distribution. Is it just a sampling bias? This is important as their 1.5 month data in Figure 3-1 again show no statistical difference for the mean, but highly significant differences using the KS test. This should be discussed.

5. Related to the above point, the 1.5 month data show no differences for either the frequency or amplitude of mEPSCs between WT and Gde2 KO neurons. They also found no changes in NMDAR mediated LTD at 1.5 months (Fig. 4-2). This raises further concern that any effects seen at 7 months are secondary to the effects of GDE2 on neuronal survival. If GDE2 plays a role in synaptic function, it should be detectable at 1.5 months.

6. The authors detected significant activation of the PI3K-Akt-GSK3beta pathway in the Gde2KO mice at 7 months. However no significant differences was detected at 1.5 months. This again suggests that the effects seen at 7 months are secondary. Since many physiological and pathological stressors induce Akt activation, this further raises concerns that the Akt activation is a secondary response to the effects of Gde2 on neuronal survival.
