# Supplementary figures and images for: Novel Roles of the GPI-Anchor Cleaving Enzyme, GDE2, in Hippocampal Synaptic Morphology and Function
Source: eNeuro. 2025 Jul 23;12(7):ENEURO.0102-25.2025. doi: 10.1523/ENEURO.0102-25.2025 (PMC12320762; doi:10.1523/ENEURO.0102-25.2025)

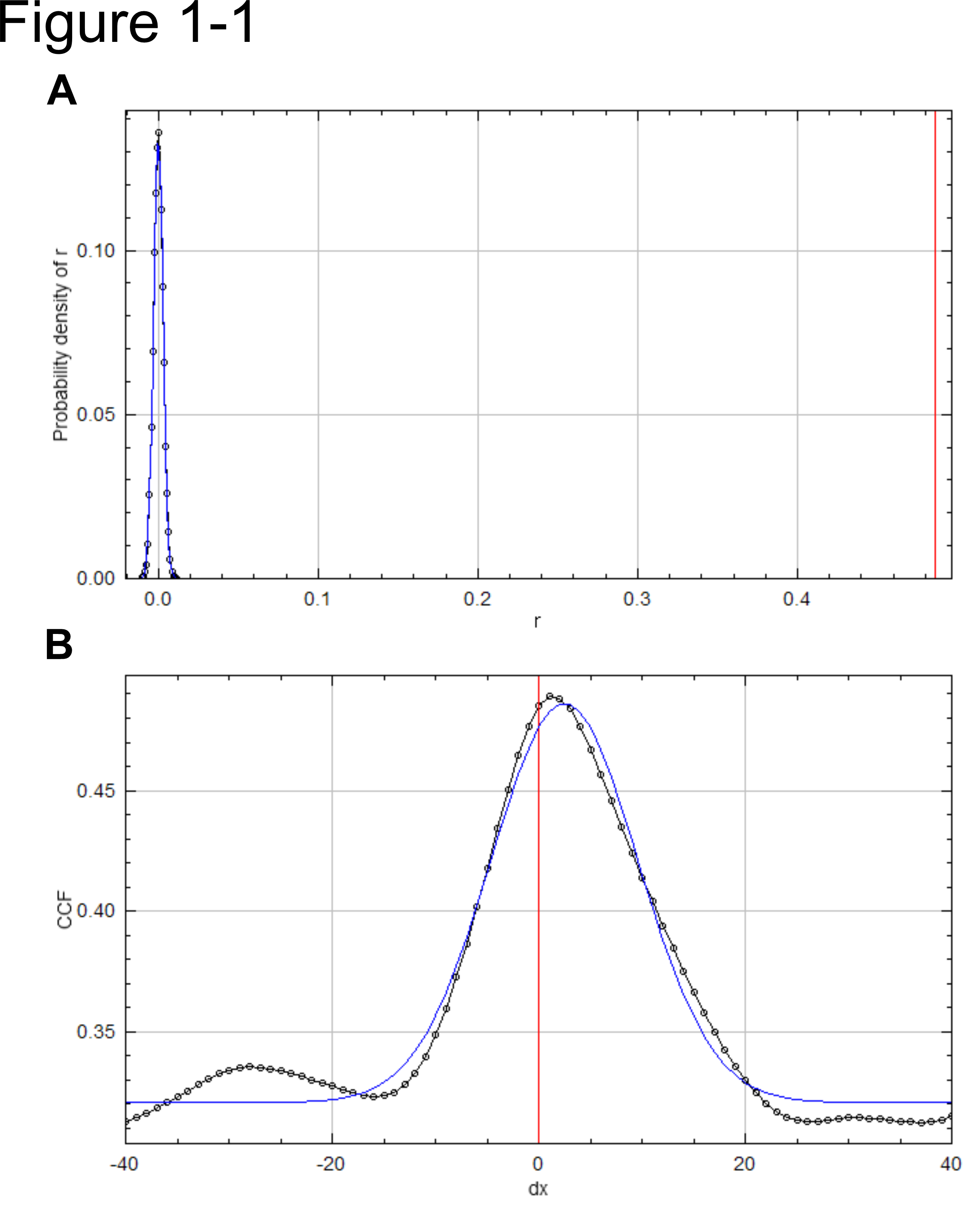

Supplement: Figure 1-1 — Colocalization randomization and x-shift test representative data Representative graphs generated from Bassoon/GDE2-Flag images from Figure 1F. For both A and B, a gaussian curve is fitted to the data. (A) Coste’s Method: Plotted data (Black dots) and fitted model (Blue curve) of the distribution of Pearson’s Coefficients (PC, denoted as r) between Bassoon and GDE2-Flag for a single representative z-stack of a neurite, obtained by scrambling the particles in the image 10,000 times, along with the measured Pearson’s Coefficient from the original image (Red line). In this case, the distribution shows that the probability of obtaining the measured PC (Red line) randomly is extremely small, meaning this colocalization is significantly above chance, and therefore can be used in analysis. (B) Van Steensel’s x-shift: Plotted data (Black) and fitted model (Blue curve) of the cross-correlation function (CCF, as measured by PC) between Bassoon and GDE2-Flag for a single representative z-stack of a neurite as a function of the pixel shift (dx) between the stains in the positive and negative direction. The red line delineates the PC in the original image. As expected, a larger pixel shift between the stains leads to a smaller correlation, indicating that our measured PC is unique. Download Figure 1-1, TIF file. [file eneuro-12-ENEURO.0102-25.2025-s002.tif]

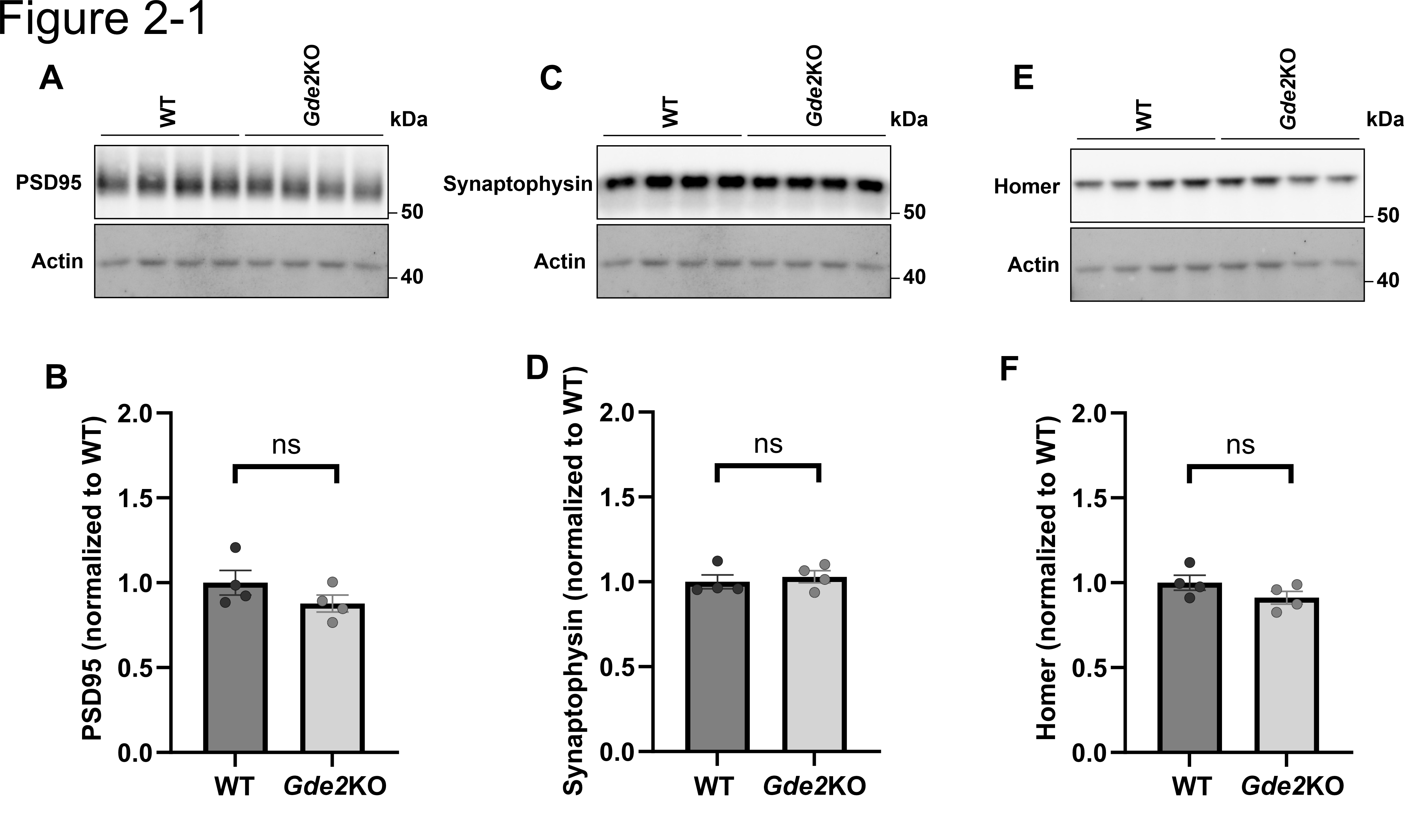

Supplement: Figure 2-1 — GDE2 does not regulate hippocampal synaptic protein amounts in 7-month mice. (A, C, E) Western blots of 7-month WT and Gde2KO hippocampal extracts (n = 4 animals per genotype, unpaired t test). Actin is used as a loading control. (A) PSD95 (C) Synaptophysin (E) Homer. (B, D, F) Graphs quantifying western blots normalized to Actin prior to normalizing to WT for (B) PSD95 (ns p = 0.2126), (D) Synaptophysin (ns p = 0.6036), and (F) Homer (ns p = 0.1765). (B, D, F) All graphs: mean ± s.e.m.. See Table 1 for statistical summaries. Download Figure 2-1, TIF file. [file eneuro-12-ENEURO.0102-25.2025-s003.tif]

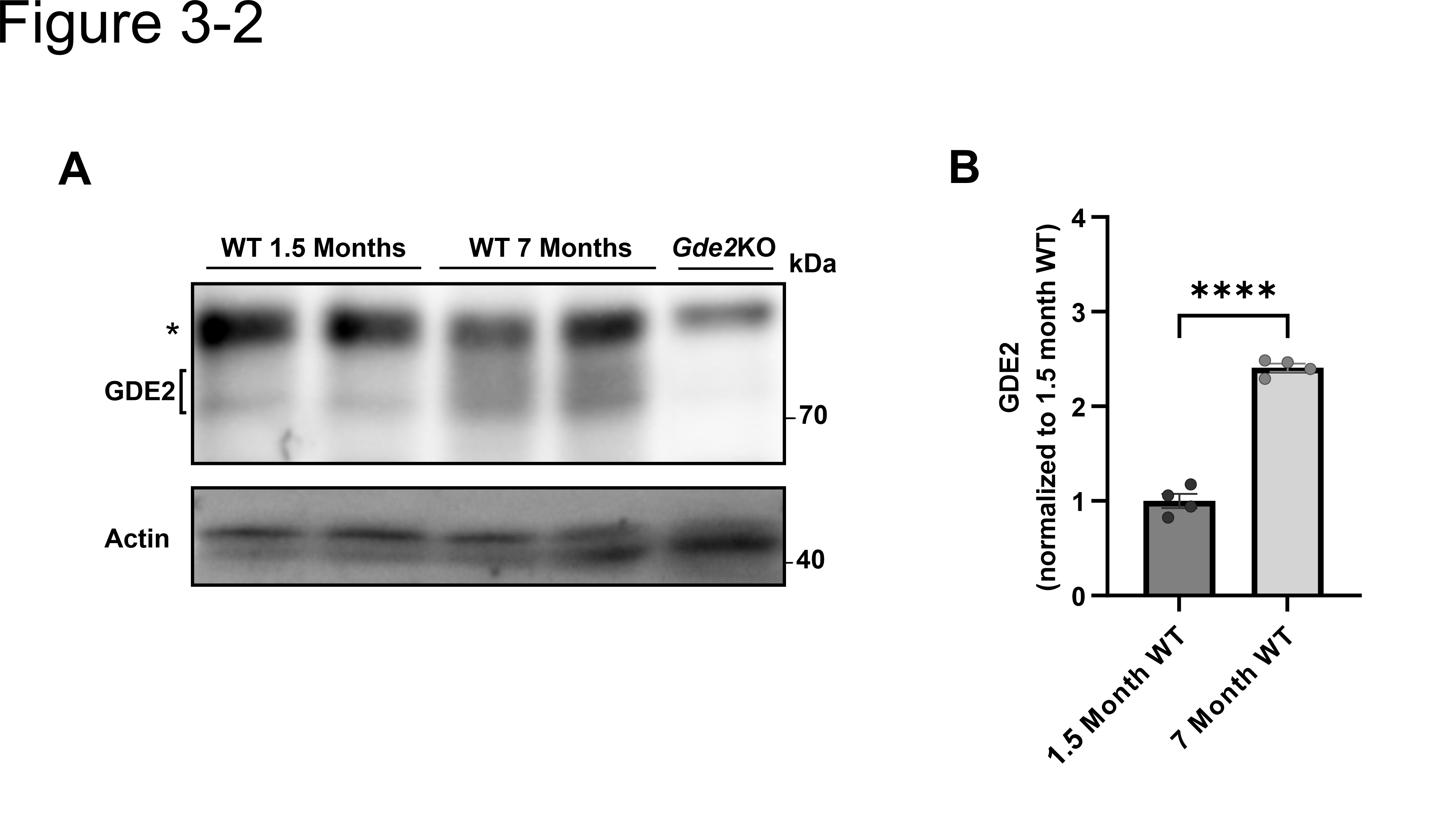

Supplement: Figure 3-2 — GDE2 loss does not significantly impact mEPSC properties or frequency/amplitudes at 1.5-months. (A-D) Waveform kinetics and resistance measurements of mEPSCs from 7-month WT and Gde2KO CA1 cells (n = 11 WT cells, n = 10 Gde2KO cells, 4 animals per genotype, unpaired t-test). Rise time (A, ns p = 0.5239) decay tau (B, ns p = 0.9496), series resistance (C, ns p = 0.6225), and membrane resistances (D, ns p = 0.7059). (E-N) Whole-cell patch-clamp mEPSC recording data from 1.5-month-old Gde2KO and WT CA1 pyramidal cells (n = 10 WT cells, n = 11 Gde2KO cells, 5 animals per genotype, all bar graphs, unpaired t-test). (E) Superimposed representative averaged traces aligned by the start of rise time for each genotype (x-axis: 5 msec, y-axis: 5 pA). (F) Graph quantifying the mean amplitude of events for both genotypes (ns p = 0.0790). (G) Cumulative distribution of amplitudes, with dashed line showing 50% cumulative probability (Kolmogorov-Smirnov test, ****p < 0.0001). (H) 30-second representative raw recording traces (x-axis: 5 sec, y-axis: 10pA). (I) Graph quantifying the mean frequency of events for Gde2KO and WT cells (ns p = 0.0949). (J) Cumulative distribution of event intervals, with dashed line showing 50% cumulative probability (Kolmogorov-Smirnov test, ****p < 0.0001). (K-N) Waveform kinetics and resistance measurements of mEPSCs. Rise time (K, ns p = 0.2225), decay tau (L, ns p = 0.9490), series resistance (M, ns p = 0.4825), and membrane resistances (N, ns p = 0.4013). All bar graphs: mean ± s.e.m.. See Table 1 for statistical summaries. Download Figure 3-2, TIF file. [file eneuro-12-ENEURO.0102-25.2025-s005.tif]

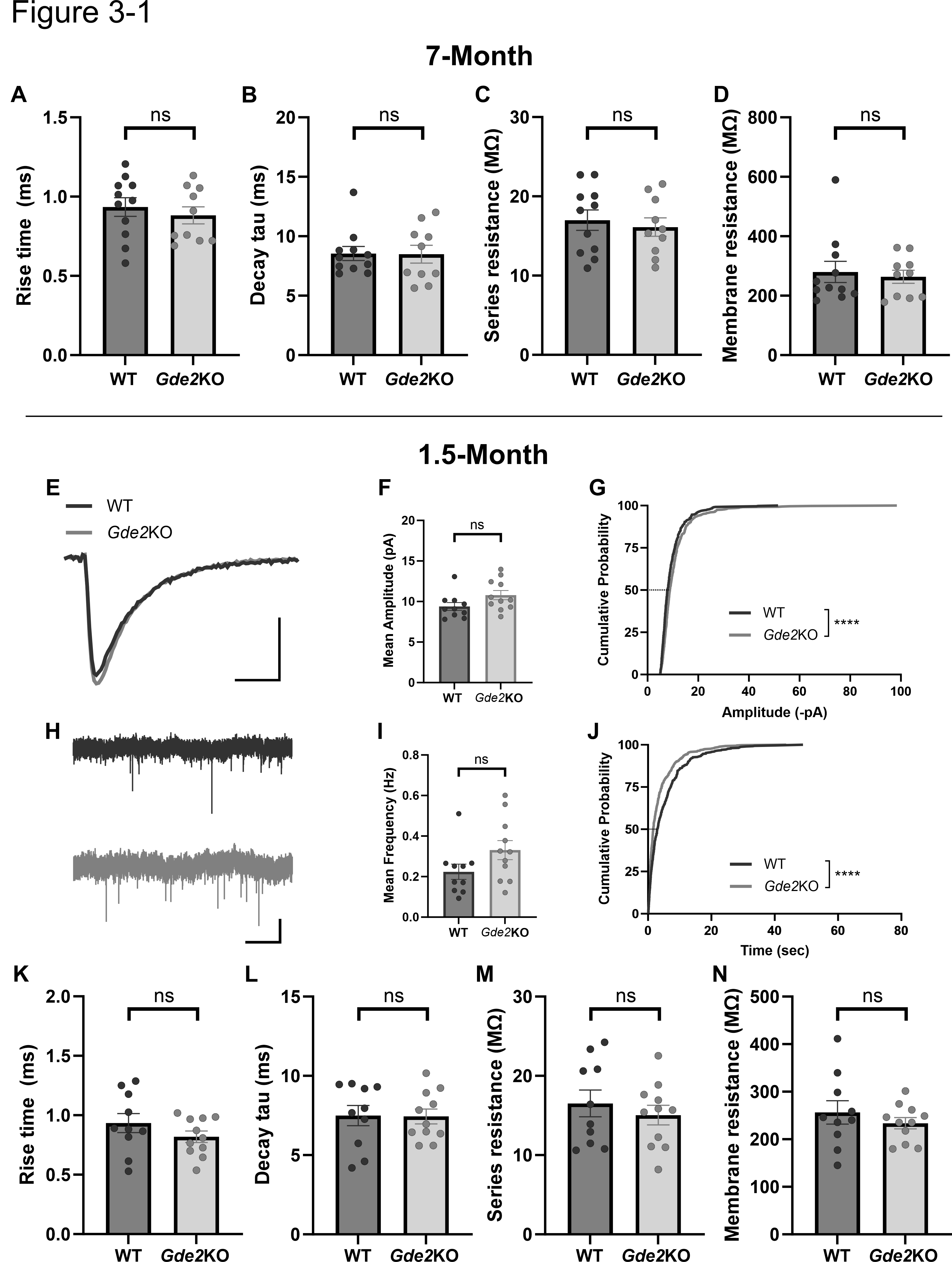

Supplement: Figure 3-1 — GDE2 hippocampal expression is higher at 7-months compared with 1.5-months. (A) Representative western blot of hippocampal lysates from 1.5- and 7-month WT mice probing for GDE2 and Actin (n = 4 animals per time point). GDE2 (bracket) typically runs as a series of diffuse bands due to glycosylation; accordingly, Gde2KO hippocampal lysate run on the same gel is referenced as a control. The asterisk denotes a non-specific protein band. (B) GDE2 levels are 2.4 fold higher at 7 months compared to 1.5 months after normalization to actin (mean ± s.e.m., unpaired t test, p**** < 0.0001). Download Figure 3-1, TIF file. [file eneuro-12-ENEURO.0102-25.2025-s004.tif]

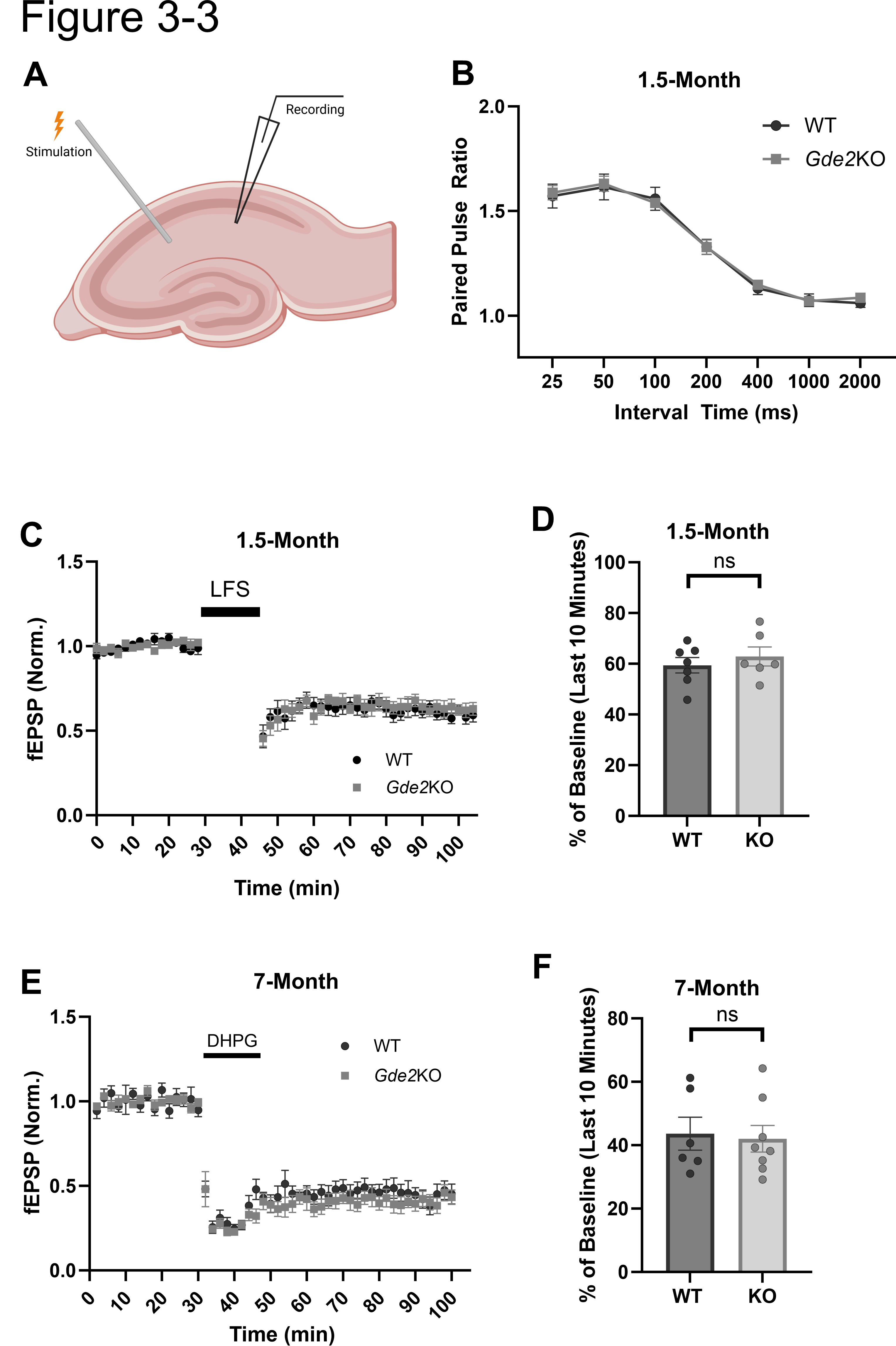

Supplement: Figure 3-3 — GDE2 does not impact 1.5-month PPR, NMDAR-dependent LTD, or 7-month mGluR-dependent LTD. (A) Schematic of CA1 hippocampal slice field recording set up showing stimulation and recording electrode placement in the stratum radiatum. (B) Paired-pulse facilitation (PPF) induction of CA1 field EPSPs (fEPSPS) in 1.5-month Gde2KO and WT slices (n = 10 WT slices from 5 animals, n = 12 Gde2KO cells from 5 animals per genotype) reported as the ratio of the second fEPSP slope to the first fEPSP slope (y-axis) tested using various interstimulus interval (ISI) between the first and second pulses (25, 50, 100, 200, 400, 1000, and 2000 msec). (C and D) LFS-induced NMDAR-dependent LTD in Gde2KO and WT slices at 1.5-months (C, n = 7 WT slices, n = 6 Gde2KO cells, 4 animals per genotype) reported as the ratio of the induced response’s fEPSP slope to the average baseline fEPSP slope (first 30 minutes) and graph quantifying the average fEPSP slope during the last 10 minutes of recording data as a percent of the average fEPSP slope during baseline for both genotypes (D, ns p = 0.4783, unpaired t test). (E and F) DHPG-induced mGluR-dependent LTD in Gde2KO and WT slices at 7-months (E, n = 6 WT slices, n = 8 Gde2KO cells, 3 animals per genotype) reported as the ratio of the induced response’s fEPSP slope to the average baseline fEPSP slope (first 30 minutes) and graph quantifying the average fEPSP slope during the last 10 minutes of recording data as a percent of the average fEPSP slope during baseline for both genotypes (F, ns p = 0.8145, unpaired t test). (D and F) All bar graphs: mean ± s.e.m.. Schematic in A created in BioRender.com. See Table 1 for statistical summaries. Download Figure 3-3, TIF file. [file eneuro-12-ENEURO.0102-25.2025-s006.tif]

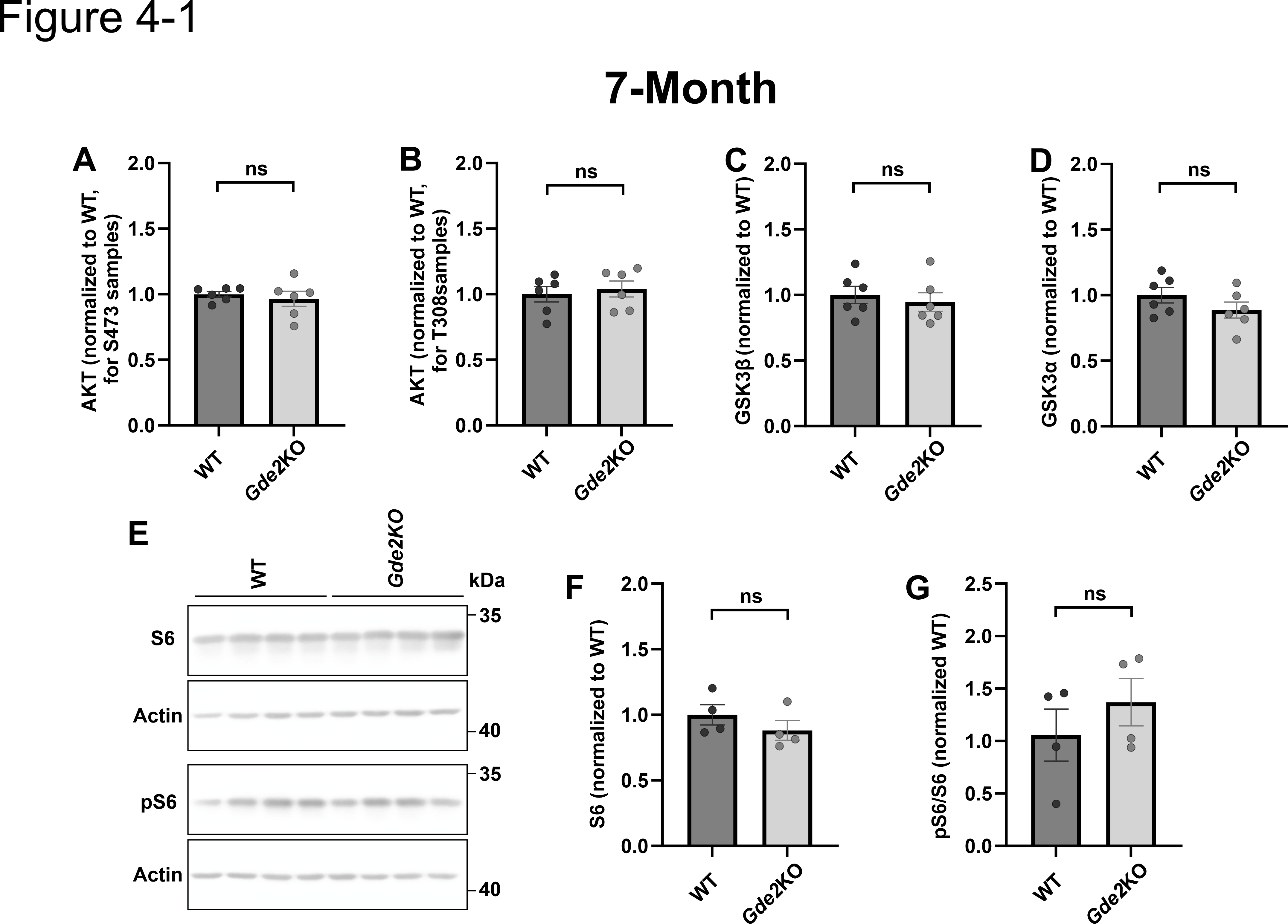

Supplement: Figure 4-1 — GDE2 does not regulate total AKT, GSK3, S6, and phosphorylated S6 in 7-month hippocampus. (A-D) Graphs quantifying western blots from 7-month WT and Gde2KO hippocampal extracts (n = 6 animals per genotype, unpaired t test) normalized to Actin prior to normalizing to WT. See main Figure 4 for blots. (A and B) AKT (AKT (S473) blot: ns p = 0.5782, AKT (T308) blot: ns p = 0.6481), (C) GSK3β (ns p = 0.5931), and (D) GSK3ɑ (ns p = 0.2083). (E) Western blots of 7-month WT and Gde2KO hippocampal extracts (n = 4 animals per genotype) for S6 and phosphorylated S6. Actin is used as a loading control. (F and G) Graphs quantifying western blots normalized to Actin prior to normalizing to WT for (F) S6 (ns p = 0.3130) and (G) phosphorylated S6 (ns p = 0.3850), unpaired t test. All graphs: mean ± s.e.m.. See Table 1 for statistical summaries. Download Figure 4-1, TIF file. [file eneuro-12-ENEURO.0102-25.2025-s007.tif]

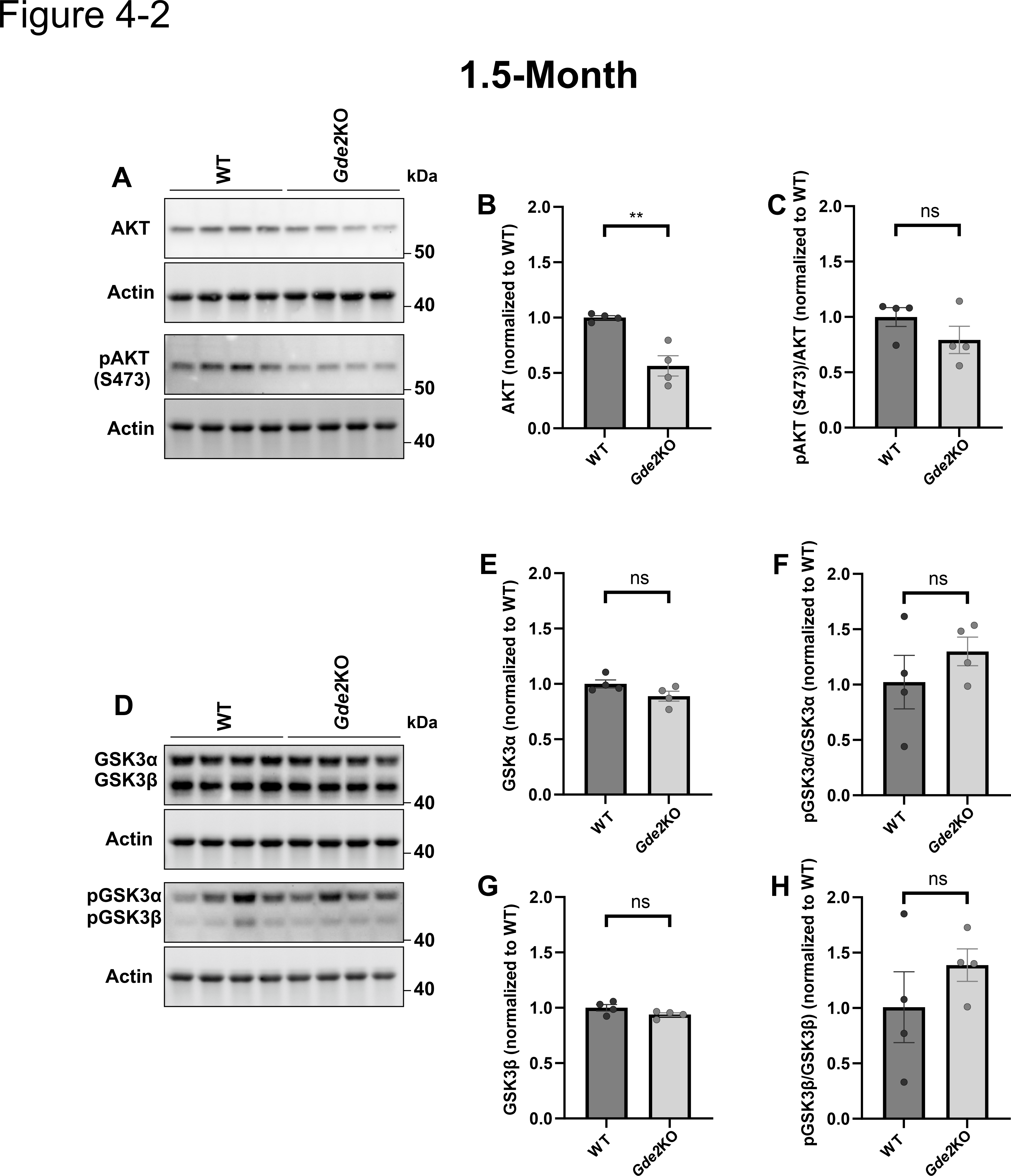

Supplement: Figure 4-2 — GDE2 does not modulate hippocampal phosphorylated AKT and GSK3 in 1.5-month mice. (A, D) Western blots of 1.5-month WT and Gde2KO hippocampal extracts (n = 4 animals per genotype). Actin is used as a loading control. (A) total and phosphorylated AKT (S473), (D) total and phosphorylated GSK3β and GSK3ɑ. (B, C, E-H) Graphs quantifying western blots normalized to Actin prior to normalizing to WT for (B) total AKT (**p = 0.0033), (C) phosphorylated AKT (S473) to total AKT (ns p = 0.2173), (E) total GSK3ɑ (ns p = 0.1375), (F) phosphorylated GSK3ɑ to total GSK3ɑ (ns p = 0.3509), (G) total GSK3β (ns p = 0.1155), (H) phosphorylated GSK3β to total GSK3β (ns p = 0.3224). All graphs: mean ± s.e.m., unpaired t test. See Table 1 for statistical summaries. Download Figure 4-2, TIF file. [file eneuro-12-ENEURO.0102-25.2025-s008.tif]

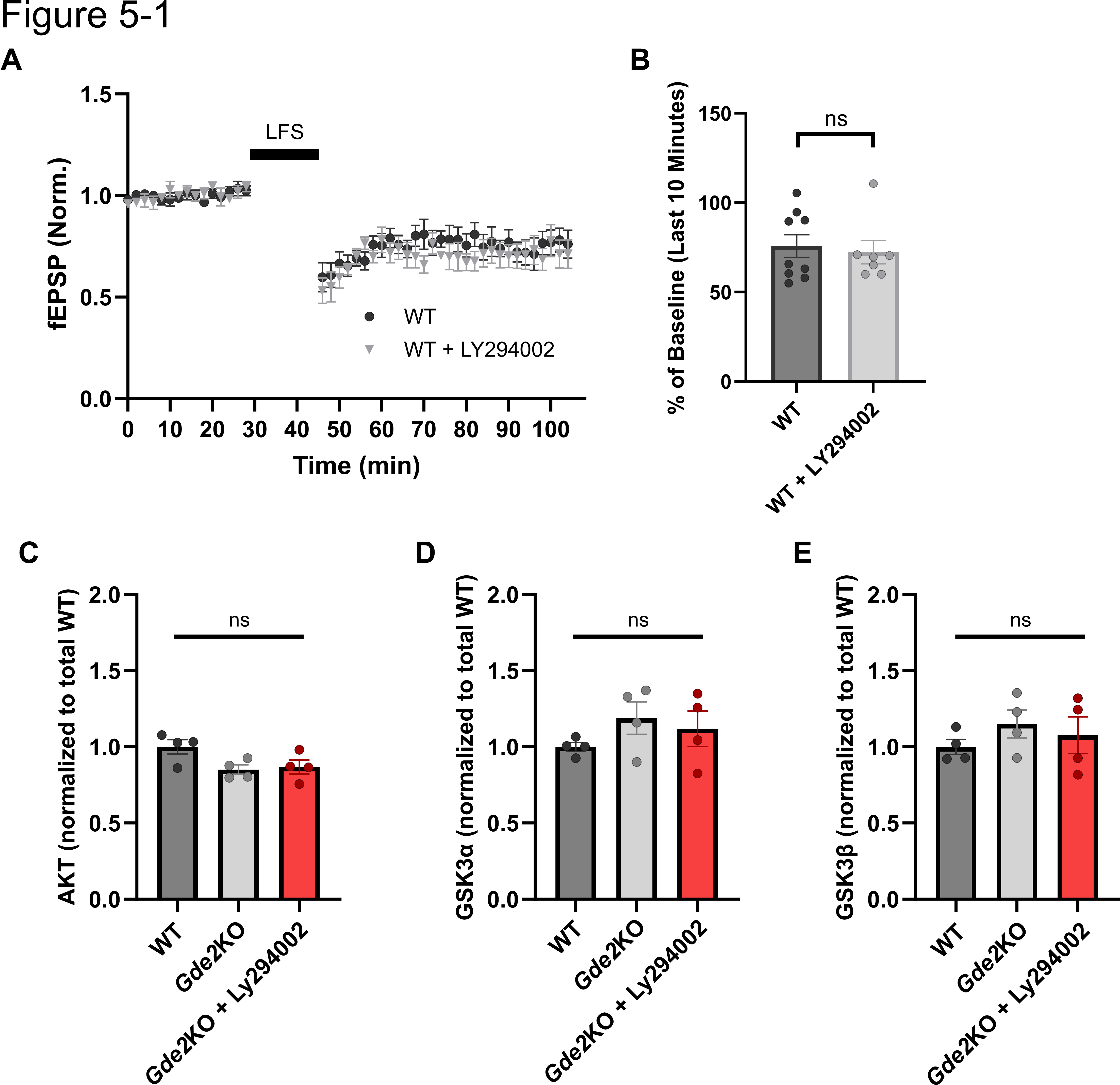

Supplement: Figure 5-1 — LY294002 does not reduce WT NDMAR-dependent LTD or affect total AKT and GSK3 levels in Gde2KO slices (A) LFS-induced NMDAR-dependent LTD in slices from 7-month WT and WT with LY294002 applied, reported as the ratio of the induced response’s fEPSP slope to the average baseline fEPSP slope (first 30 minutes, WT data from Fig. 3, n = 7 slices from 5 animals for WT + LY294002 condition). (B) Graph quantifying the average fEPSP slope during the last 10 minutes of recording data as a percentage of the average fEPSP slope during baseline (WT data from Fig. 3, unpaired t test, ns p = 0.7193). (C – E) Graphs quantifying western blots from 7-month WT, Gde2KO, and Gde2KO slices treated with LY294002 (n = 4 animals per condition) normalized to Actin prior to normalizing to WT for total AKT (C, one-way ANOVA, ns p > 0.05), GSK3α (D, one-way ANOVA, ns p > 0.05) and pGSK3β (E, one-way ANOVA, ns p > 0.05). All bar graphs: mean ± s.e.m.. See Table 1 for statistical summaries. Download Figure 5-1, TIF file. [file eneuro-12-ENEURO.0102-25.2025-s009.tif]
